# Stakeholders′ Perceptions of Environmental and Public Health Risks Associated with Hydrocarbon Activities in and around the Vasilikos Energy Center, Cyprus

**DOI:** 10.3390/ijerph182413133

**Published:** 2021-12-13

**Authors:** Eleni G. Kleovoulou, Corina Konstantinou, Andria Constantinou, Eelco Kuijpers, Miranda Loh, Karen S. Galea, Rob Stierum, Anjoeka Pronk, Konstantinos C. Makris

**Affiliations:** 1 Cyprus International Institute for Environmental and Public Health, Cyprus University of Technology, Limassol 3041, Cyprus; eg.kleovoulou@edu.cut.ac.cy (E.G.K.); corina.konstantinou@cut.ac.cy (C.K.); andriaconstantinou95@gmail.com (A.C.); 2 The Netherlands Organization for Applied Scientific Research TNO, 3584 CB Utrecht, The Netherlands; eelco.kuijpers@tno.nl (E.K.); rob.stierum@tno.nl (R.S.); anjoeka.pronk@tno.nl (A.P.); 3 Institute of Occupational Medicine (IOM), Edinburgh EH14 4AP, UK; miranda.loh@iom-world.org (M.L.); karen.galea@iom-world.org (K.S.G.)

**Keywords:** public perceptions, environmental health, community risks, industrially contaminated sites, oil and gas, hydrocarbons, petroleum, exposome, stakeholder consultation, thematic analysis

## Abstract

The Vasilikos Energy Center (VEC) is a large hydrocarbon industrial hub actively operating in Cyprus. There is strong public interest by the communities surrounding VEC to engage with all stakeholders towards the sustainable development of hydrocarbon in the region. The methodological framework of the exposome concept would allow for the holistic identification of all relevant environmental exposures by engaging the most relevant stakeholders in industrially contaminated sites. The main objectives of this study were to: (i) evaluate the stakeholders’ perceptions of the environmental and public health risks and recommended actions associated with the VEC hydrocarbon activities, and (ii) assess the stakeholders’ understanding and interest towards exposome-based technologies for use in oil and gas applications. **Methods:** Six major groups of stakeholders were identified: local authorities, small-medium industries (SMIs) (including multi-national companies), small-medium enterprises (SMEs), academia/professional associations, government, and the general public residing in the communities surrounding the VEC. During 2019–2021, a suite of stakeholder engagement initiatives was deployed, including semi-structured interviews *(n = 32*), a community survey for the general public (*n* = 309), technical meetings, and workshops (*n = 4*). Results from the semi-structured interviews, technical meetings and workshops were analyzed through thematic analysis and results from the community survey were analyzed using descriptive statistics. **Results:** Almost all stakeholders expressed the need for the implementation of a systematic health monitoring system for the VEC broader area and its surrounding residential communities, including frequent measurements of air pollutant emissions. Moreover, stricter policies by the government about licensing and monitoring of hydrocarbon activities and proper communication to the public and the mass media emerged as important needs. The exposome concept was not practiced by the SMEs, but SMIs showed willingness to use it in the future as part of their research and development activities. **Conclusions:** The sustainable development of hydrocarbon exploitation and processing prospects for Cyprus involves the VEC. Continuous and active collaboration and mutual feedback among all stakeholders involved with the VEC is essential, as this may allow future environmental and occupational health initiatives to be formalized.

## 1. Introduction

The process of understanding public perceptions about environmental health hazards, exposures, and health impacts often feeds policy making and decision support systems of public health [1,2]. Residents’ perceptions about environmental hazards in and around industrial contaminated areas (ICS) have always attracted attention [2,3,4,5,6,7,8,9]. For example, the top three environmental issues of concern that may affect respondents’ health in ICS in the USA were chemicals in consumer products, outdoor air quality, and drinking water quality, while the most frequently perceived health issues were respiratory illness, asthma, and cancer [2]. Similarly, pollution and health effects, followed by reliability, damage to nature, and cost, were among the greatest concerns of USA residents towards solid waste management facilities [6].

Government agencies and industries are often responsible for communicating environmental risk information to the public regarding, for example, regulatory decisions, industrial practices, or adverse events [4]. Another study [10] addressed the safety and environmental problems in the petroleum industry of Nigeria. The authors examined factors within the wider socio-political and governance context, going beyond the regulatory framework system that often impacts safety and environmental quality aspects. Their key contributing factors were the poor governance, rent seeking culture, and inadequate funding, all interfacing with the financial and governmental context. In the same context, one study [11] investigated the socio-cultural preferences of stakeholders in the Niger Delta to understand how different stakeholder groups value socio-cultural differences. Based on their results, all stakeholders acknowledged the issue of hydrocarbon pollution and its impact on water and health. Moreover, community members, regulators, experts, and operators similarly valued water quality, soil quality, and food production. As it is observed by the current manuscript authors, there is citizen involvement and intention to co-create approaches and activities that engage all stakeholders impacted by environment and health concerns 

There is a need for a comprehensive methodological framework that would allow for the holistic capture of all relevant environmental exposures in and around ICS by engaging the most relevant stakeholders. The ‘human exposome’ concept was first proposed by Wild (2005) to encompass the totality of human environmental (meaning all non-genetic) exposures from conception onwards, complementing the genome. Implementing the human exposome methodological framework and describing variations in the exposome might allow for a thorough assessment of the entirety of environmental changes associated with ICS activities [12]. The observable amount of the exposome is a biological indicator of our upbringing and the setting in which various exposures have an influence on health [12,13,14]. The totality of this exposome concept is anticipated to aid developments towards the better and more comprehensive characterization of environmental and public health risks around ICS via proper channeling and knowledge exchange with all relevant stakeholders.

**Figure 1:**
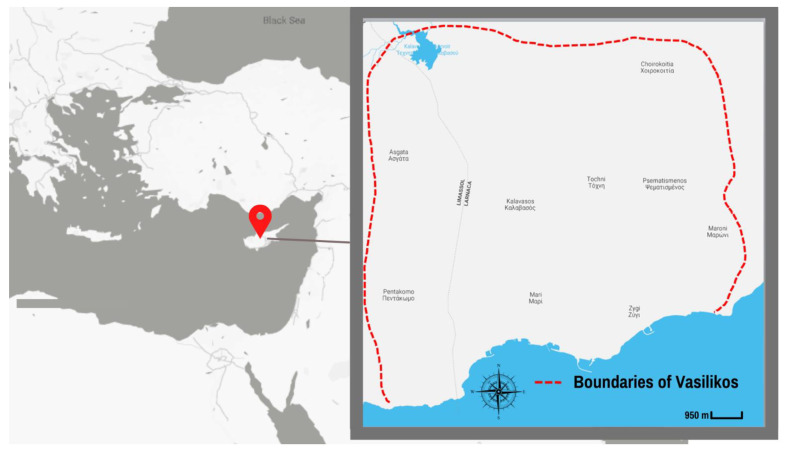
The Vasilikos Energy Center and its surrounding area, 5–10 km from the nearby city Limassol. The VEC industrial hub is situated on the coastline within the red dotted perimeter of the map and located on the south coast of Cyprus and within the broader eastern Mediterranean region. The residential communities around the VEC industrial hub are also located within the red dotted perimeter.

The Vasilikos Energy Center (VEC) area is a coastal heavy industry zone in Cyprus, close to the seaside area of Limassol (Figure 1). Significant quantities of natural gas were discovered around the end of 2011 in Plot 12 of the Cyprus exclusive economic zone (EEZ) [15]. The natural gas reserves found in the EEZ of Cyprus are considered the largest investment for future generations of modern Cypriot society, while the 2014–2020 Cyprus Smart Specialization Strategic Plan placed energy-related research initiatives at the top of the national priority list. In order to increase the autonomy and energy independence of the southern European region, the EEZ region is of primary importance. However, given the expansion of the oil and gas sector in Cyprus, there is also a need to similarly increase the capacity of the occupational and environmental health sciences to maintain active surveillance of affected communities. As a result, an updated master plan of the Vasilikos area has been prepared by discussing the prospects of developing a framework that would enable optimum industrial development [16]. The Strategic Environmental Impact Assessment examined the updated master plan of the Vasilikos area, which also aimed for a possible extension of the liquefied natural gas plant, the oil and liquefied petroleum gas facilities, and the further development of the industrial area for future gas/oil operations [15]. The sustainable development of the VEC site warrants the active engagement of all stakeholders to ensure the co-creation of opportunities and processes to protect the environment and public health of workers, the surrounding communities, and the ecosystems in place.

The growth of VEC in Cyprus’ seaside area and its impact on surrounding communities’ health can be better understood by employing a comprehensive framework, such as the human exposome. As a result, the exposome of the population around the VEC is defined by habitual patterns, as well as the context in which they occur, the accompanying environmental exposures, and personal and contextual factors, such as occupational factors or individual behaviors and lifestyle habits [12,13,17]. In this study, we explored the interest and the knowledge of the exposome concept among the research-oriented stakeholders.

According to the Cyprus Department of Environment, the VEC development in the area is expected to impact the flora and fauna biodiversity and the natural and marine landscape, as well as human health and cultural/archaeological heritage. Adverse impacts are also anticipated on water and soil resources, including waste accumulation; however, these can be managed through the implementation of mitigation measures [15]. As a result, the main objectives of this study were to: (i) evaluate stakeholders’ perceptions of environmental and public health risks associated with the hydrocarbon activities in the VEC and (ii) assess stakeholders’ understanding, acceptance, and interest towards the application(s) of exposome-based technologies, given that the VEC is surrounded by several residential communities at a short distance (1–20 km away).

## 2. Methods

### 2.1. Setting

The study setting was the active industrial hub for hydrocarbons located in the Vasilikos area of Cyprus (Figure 1). Currently, there are multiple industrial facilities located in the Vasilikos area, such as, a power plant, which is the largest of the three power plants on the island, a cement works with its own port, a large oil filling station operator, a naval base, and an oil storage terminal. In addition, several quarries and a clays/limestone processing facility are based in the area [16].

### 2.2. Stakeholders

Stakeholders were identified by searching in relevant contact databases (e.g., Cyprus International Institute for Environmental and Public Health, Cyprus University of Technology) and by contacting governmental departments and relevant companies in the territories of the Republic of Cyprus. A list of 45 stakeholders was created, with due consideration being given to the General Data Protection Regulation 2018 and the project data management plan. As a result, we grouped all identified stakeholders into six distinct groups (the number of organizations that agreed to participate in this exercise is included in the parentheses):

A. **Government (GOV)** (*n = 9*)**:** governmental departments and units responsible for hydrocarbons’ authorizations, strategic planning, and energy provision.
B. **Small-medium industries (SMIs)***(n = 6*): companies which are actively involved in Cyprus with activities of exploration, production, management and distribution of hydrocarbons, both onshore and offshore; large multi-national oil and gas companies are also included in this group.
C. **Small-medium enterprises (SMEs)** (*n = 4*): companies related with the hydrocarbon industry of Cyprus, including environmental consultants, laboratories, and engineers.
D. **Academia/professional associations (APA)** (*n = 4*)**:** academics and researchers who are involved in hydrocarbon research.
E. **Local residential community authorities (LA)** (*n = 9*)**:** Local authorities of the surrounding areas of Vasilikos and, specifically, leaders of the nine communities in the area: Asgata, Pentakomo, Tochni, Kalavasos, Choirokitia, Mari, Maroni, Zygi, and Psematismenos.
F. **Public/Communities** (*n* = 3700 adults, based on the 2011 CENSUS survey): residents of the nine communities around the Vasilikos (VEC) area.

### 2.3. Engagement Initiatives

A series of engagement initiatives were employed, ranging from face-to-face and virtual meetings and interviews to workshops and community surveys. The exposome concept was always presented first in these meetings and interviews, by offering its definition and practical applications, as well as case studies that were relevant to their professional field.

### 2.4. Stakeholders’ Workshops and Local Authorities’ Meetings

Stakeholder workshops were held bi-annually and were open for all interested stakeholders. Their purpose was to allow stakeholders to provide their views on the specific workshop’s topics of discussion. Moreover, two meetings with the nine communities’ leaders in the Vasilikos area took place to present the aim of the study, to discuss the population survey implementation, and to explain the purpose of the stakeholder interviews, as well as to understand their views and concerns. A final workshop was also arranged in September 2021 to communicate the results of the stakeholder engagement process and to finalize the next steps based on the recommendations of the engagement process.

### 2.5. Semi-Structured Interviews

Semi-structured telephone interviews using a core set of questions were chosen as the most appropriate approach for the 32 stakeholders who participated in the study (S1), except for the public. The conducted interviews contained questions that focused on health risks about the hydrocarbon activities in the Vasilikos area [18,19,20] and were tailored for each stakeholder group. The interviews took place during two different time periods, these being October–November 2019, with representatives from the GOV, SMIs, SMEs, and APA (*n* = *23*), and in October 2020, with representatives of the LA of Vasilikos area (*n* = *9*). The interviews ranged from 15–45 min in length. The data collected from the interviews were recorded and transcribed verbatim.

### 2.6. Community Survey

The perceptions of the general public residing in the communities around the VEC area were evaluated with the use of a postal survey based on validated questionnaires [21,22,23]. The questionnaire was administered in paper format and written informed consent was obtained. Specifically, it examined the opinions of the participants regarding their relationship with the environment, assessed their beliefs about the environment of their community and health-related risks, and gathered information regarding the residents’ lifestyle (i.e., physical activity, eating habits, smoking status, etc.), health status, and demographic characteristics (S1–S4).

Eligible participants were adults (>18 years old) living in the residential communities surrounding the VEC industrial hub, i.e., Asgata, Pentakomo, Tochni, Kalavasos, Choirokitia, Mari, Maroni, Zygi, and Psematismenos, for at least 1 year (permanent residents). The questionnaires were sent via mail to the adult population of Vasilikos area (3700 adults, based on the 2011 CENSUS survey for the nine communities), directly from the main office of the respective community councils, in early August 2020. The local authorities notified the residents about the study through the communities’ postal system before the questionnaires were distributed in order to encourage them to participate. The completed questionnaires were returned and received in sealed postal envelopes and were handled based on the General Data Protection Regulation (GDPR) of the European Union.

## 3. Data Analysis

### 3.1. Interviews, Workshops and Meetings

A qualitative research protocol was implemented as it explores complex phenomena encountered by clinicians, health care providers, policy makers and consumers in health care [24,25]. Qualitative research aims to convey a global perception of reality based on rich and detailed elements that appear in their natural social context, rather than to create superficial models, trends, and correlations [24]. The interviews, workshops and meetings transcripts were interpreted with the aid of thematic analysis [25,26,27]. Thematic analysis is a versatile and effective research approach that can produce a comprehensive, detailed, and complex explanation of data, including the assessment of how events, realities, meanings, experiences, and so on are influenced by a variety of societal discourses [28]. In contrast to other methods such as grounded theory, discourse analysis, and so on, thematic analysis is not bound by any pre-existing theoretical framework and can thus be used within different theoretical frameworks [28]. A list of codes was produced, representing themes identified in the textual data (S3). After the coding stage, a list of different codes was created and used to search for themes [28]. Each code was given a heading and all text relevant to that category was stored under that heading, thus creating a broad subset [25,26,27]. Next, the codes were analyzed and combined to form an overarching theme [28].

### 3.2. Community Survey

Descriptive statistics were used to analyze the questionnaire responses by 309 residents. The last open-ended question of the questionnaire was analyzed through thematic analysis. For the rest of the close-ended questions, means and standard deviation for the continuous variables, and frequencies and percentages by category for the categorical variables, were calculated and reported. All analyses were conducted in R 4.0.2 with RStudio 1.1.463 [29,30]. Results, raw data, script, and output are available in the S4.

### 3.3. Ethics and Personal Data Management

Written informed consent was obtained from stakeholders before participating in the semi-structured interviews, workshops, and meetings. The participants were informed that the interviews would be recorded and that the collected data would be anonymous and used only for academic purposes. Moreover, the protection of the participants’ privacy in the community survey was based on the approval by the National Bioethics Committee of Cyprus (No: 2020.01.147) and in conjunction with the written opinion of the Data Protection Officer within the Cyprus University of Technology (CUT).

## 4. Results

The results emerging from the interview and community survey data were organized according to the thematic and the statistical analysis, respectively. Combining the data, eight (8) themes emerged regarding the stakeholders′ responses; assessment of health and safety issues, safety and health risks in the hydrocarbons industry, perceived environmental risks, shared responsibility on health issues, risk communication, usage of the human exposome concept by the stakeholders, and their suggestions for improvement about the industrial area of Vasilikos.

### 4.1. Assessment of Health and Safety Issues 

The LA, SMIs, SMEs, and GOV mentioned that they regularly implement health and safety assessments that relate to their company or organization’s hydrocarbon activities. However, all the above-mentioned stakeholders considered that a systematic health monitoring system studying environment and health indicators in and around the VEC industrial facilities was an important need for managing pertinent health and safety issues. They also suggested that more environmental and health studies (e.g., air and water quality measurements) must be provided in hydrocarbon facilities, especially in the VEC. Representatives from SMIs and APA mentioned:

*“With regards to the communities around the areas where we work, an initial assessment should be made of the likelihood and magnitude of the risk to have some impact from our area of activity”*

(Interview 21, SMI).

*“I believe that there is know-how and ability from the various academic departments I know. What may not exist in Cyprus, [...], are some experts in the use and management of hydrocarbons [...] such expertise probably does not exist within the academic community. There may be academic knowledge but no practical experience in the installation and operation of such facilities […] what is needed is in-depth training and experience”*

(Interview 19, APA).

### 4.2. Safety Risks in the Hydrocarbon Industry

With respect to the safety risks that employees and public may encounter due to hydrocarbon activities, various views and points were presented. The LA, APA, and the public reported that their main safety concern is the accumulation of industries at VEC, which increases the risk of an accident (e.g., explosion) with potentially devastating consequences for the area and its population. The SMIs, SMEs, and government clearly stated the use of appropriate safety and health directives in their everyday activities, while a few academics reported the need to have better implementation of the EU safety regulations in the processing and storing of oil/gas.

*“We are concerned because some permits are given, in each of the environmental studies carried out, which show that they (hydrocarbon industries) are below the permissible limits but we believe that the accumulation of many factories in the area, will result to exceedance of the permissible limits”*

(Interview 2, LA).

A representative from government mentioned that:

*“There is a plan for dealing with employees’ accidents and is national. There is no national plan for health risk management”*

(Interview 12, GOV).

### 4.3. Health Risks in the Hydrocarbon Industry

Only the LA, SMIs, and the public reported their perceived health risks associated with hydrocarbon activities. Specifically, LA and the public reported increased disease incidence in the community, such as respiratory, heart, and thyroid diseases, cancer, asthma, and depression. Similarly, the SMIs suggested that long-term exposure to several co-located hydrocarbon industries operating in a small area (VEC) possibly affects the health of employees.

*“During the last years, I am having heart problems, respiratory problems, thyroid, prostate, thymus and everything began with my return to Cyprus. It is important for me to know what is happening in this area”*

(Resident 17).

Similarly, one stakeholder representing local authorities added that:

*“Cancer occurs in too many people in our community. After all, this is the problem we are complaining about because all the factories came to our area. There are many cases of cancer in other villages (of Vasilikos area) as well”*

(Interview 3, LA).

The SMIs mentioned no emerging risks on employees’ health and that the physical and mental health of the employees is covered by the companies’ health monitoring protocols. Moreover, these stakeholders supported that there are technical and financial difficulties in conducting risk assessments and in ensuring the health of employees and public affected in the VEC. Nevertheless, SMIs, SMEs, APA, and GOV explained that epidemiological studies and health impact studies for populations around such facilities and specifically in the Vasilikos area are needed, with the last two stakeholders agreeing that cooperation between governmental departments, companies, and communities should be applied. The implementation of environmental and health protection systems was described by the industrial representatives:

*“The company has a health and safety management system. Especially in the field of health we have a separate management system that has specific provisions […] The company has an internal obligation when we do the environmental impact assessment; we analyze the possible effects on health”*

(Interview 21, SMI).

*“The basic QESH (Quality Environment Safety and Health) capital in all companies has to do with the risk and effects on the environment, health and nuisance. There is a great cost for the risk assessment […]”*

(Interview 5, SME).

### 4.4. Environmental Risks

The LA and the public presented a list of environmental risks associated with the presence of gases, garbage, chemicals, odors, dust, smoke, noise, and radiation, all associated with VEC activities. In contrast, government supported that their departments participate in various committees on health and safety issues, ensuring that these elements are secured with regards to the hydrocarbon activities in Vasilikos area. The SMIs, SMEs, and APA did not report any environmental risks caused by hydrocarbon industries, although the SMEs noticed the absence of measurements on gaseous pollutant emissions in these facilities and the necessity of their implementation to address environmental risks.

*“The biggest environmental concerns are the pollutants that will be caused by the energy center and where they will end up. After much consultation, the community here has accepted the energy center, like liquid fuels and LPG and some factories that will support them”*

(Interview 2, LA).

A governmental representative added that:

*“The development and monitoring of hydrocarbon activities take place under the Hydrocarbon Regulations, the Protocol on the Protection of the Mediterranean Sea against Pollution from the exploration and exploitation of its seabed and subsoil, and the Safety and Health Regulations offshore operations”*

(Interview 10, GOV).

### 4.5. Community Survey

**Figure 2:**
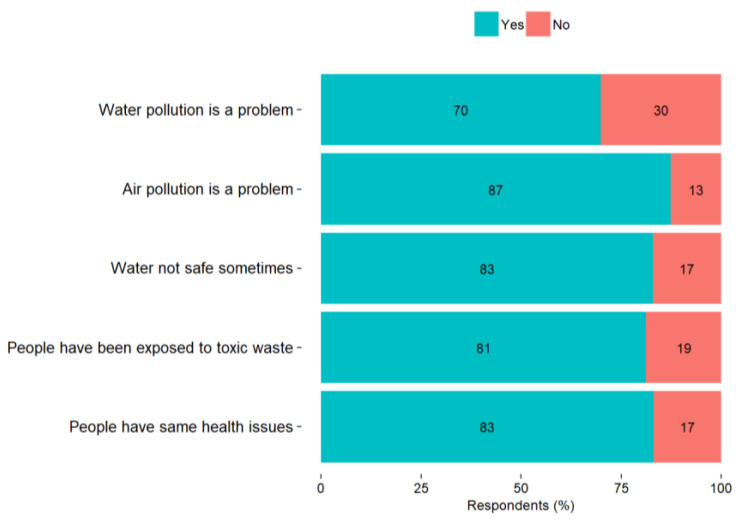
Perceptions of the respondents about their community.

The survey took place in close collaboration with the local authority offices, which were responsible for mailing the questionnaires to the households of their communities. A total of about 2700 questionnaires were mailed to the communities around the VEC. We received 309 completed questionnaires, with the response rate being around 11%. No missing data was observed in the collected questionnaire responses. About half of the respondents (residents) (51%) rated their community good or very good as a place of residence and 29% rated it as somewhat good. However, most of the respondents believe they live in a community where air and water pollution pose a problem (87% and 70%, respectively), water is not safe sometimes (87%), people have been exposed to toxic waste (81%), and have the same health issues, such as cancer, asthma, or cardiovascular diseases (83%) (Figure 2). Of those people who reported that their community has one or more of the above-mentioned environmental issues, 64% reported being very worried that this issue has harmed their health.

**Figure 3:**
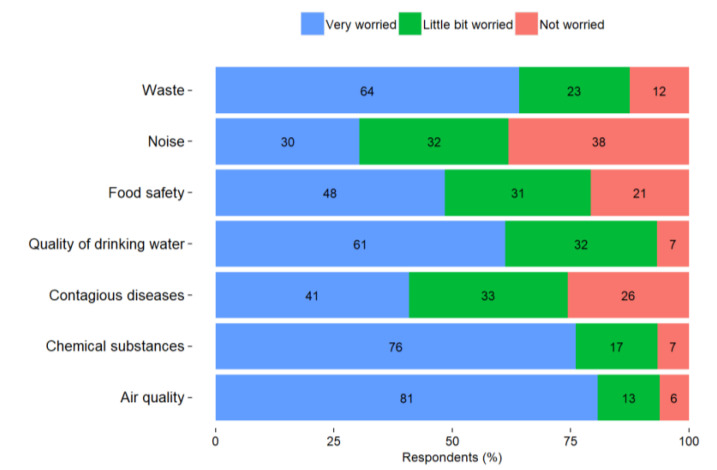
Perceived level of worry among VEC residents for environmental factors in the place of residence.

With their place of residence in mind, most of the respondents reported that they were very worried about air quality (81%), chemical substances (76%), waste (64%), and quality of drinking water (61%) (Figure 3). About half of the respondents reported being very worried about food safety (48%) and less than half about infectious diseases (41%).

During the 12 months prior to completing the survey, about 60% of the respondents reported that they have been exposed to a large degree to air pollution and bad smells, and about 70% of the respondents have been exposed to a smaller or larger degree to water pollution whilst inside or outside their home. 

**Figure 4:**
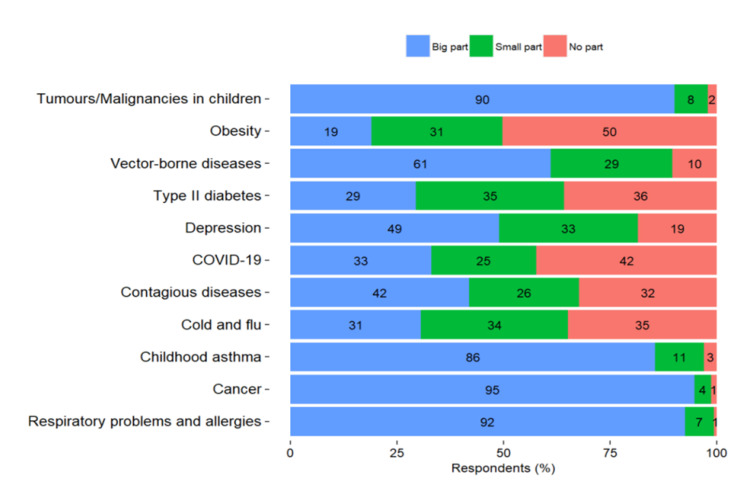
Perceived part of environmental factors in disease incidence.

Most of the participants also perceived environmental factors as playing a large part in the incidence of cancer, respiratory problems and allergies, tumors/malignancies in children, and childhood asthma (95%, 92%, 90% and 86%, respectively). More than half of the participants perceived these factors playing a large part in the incidence of vector-borne diseases (61%) and less than half in the incidence of depression and infectious diseases (49% and 42%, respectively) (Figure 4). Interestingly, half of the respondents considered that environmental factors play no part in obesity incidence and less than half in the incidence of COVID-19 and type II diabetes (42% and 36%, respectively). Furthermore, 65% of the respondents reported that they know somebody whose health has been negatively affected due to environmental factors.

### 4.6. Sharing Responsibility on Health Issues

Stakeholders do not seem to share the same views with regards to the responsibility about health issues. LA, SMEs, APA, and the public agreed that the government needs to implement stricter policies and health monitoring actions, while LA and the public reported that turning the Vasilikos area into an industrial hub was a wrong decision by the government. Furthermore, SMEs and APA added that there is incomplete knowledge and expertise from the governmental authorities on hydrocarbon issues. Alternatively, the government’s responses presented unawareness of the management of health issues, mentioning either that their department is not responsible, that other governmental departments are responsible, or that they lack of knowledge of these issues.

*“Unfortunately, all these (health issues) are due to the public service inability. They are not educated to a technical level. None of them can assess risk nor to analyze it. They don’t have the basic knowledge in order to be educated in specialized methodologies and mechanisms”*

(Interview 4, APA).

One governmental representative said that:

*“We do not know if there are any health risks associated with hydrocarbon activities that are not currently being addressed. Health issues related to occupational safety and health fall under the responsibility of the Department of Labor Inspection”*

(Interview 11, GOV).

### 4.7. Risk Communication

When asked if the public should be informed about new or current hydrocarbon activities, all stakeholders agreed that they should. SMIs mentioned that they communicate to the public before licensing of a new industry in order to alleviate misinformation and concerns; they also reported their active participation in a VEC technical committee together with the community representatives (LA), and governmental departments to oversee safety issues in the broader VEC area. However, the SMEs, APA, and the GOV added that more risk communication to the public is necessary, because natural gas is a new topic in Cyprus and public consultations and seminars could be adopted to reduce misinformation. On the element of misinformation reduction, SMEs, APA and GOV reported that mass media are part of this awareness process and the lack of mass media knowledge of hydrocarbon issues leads to wrong messages being sent to the public. As one governmental stakeholder reported:

*“People need to be informed about specific aspects of environmental issues, including hydrocarbons, if there is a reason to protect them from something. But since ordinary citizens do not have the expertise, I believe that they cannot participate when making specific decisions on health issues because unfortunately they are carried away by their semi-learning and we do not have the result we want”*

(Interview 14, GOV).

**Figure 5:**
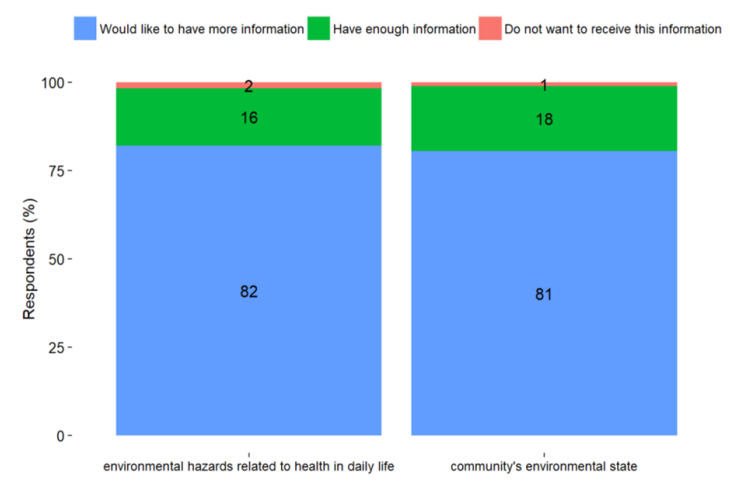
Perceived information adequacy for the environmental state of the community and environmental hazards related to health.

**Figure 6:**
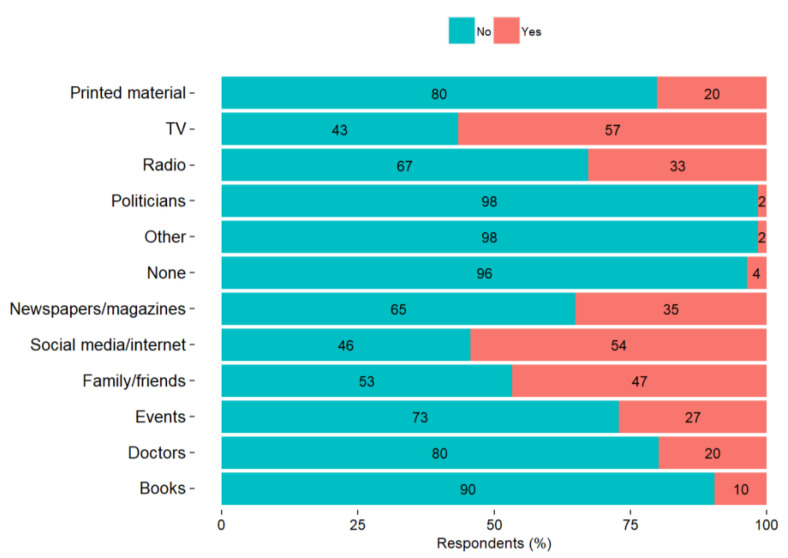
Use of information sources for environmental hazards by the residents of the communities around the VEC.

LA, as mediators of knowledge to the public, conceded that their residents are well informed by the community authorities on the environmental and public health issues that exist in the area. In general, the communities around Vasilikos area use a combination of traditional and technological methods of communication such as printed informational material, boxes of suggestions and complaints, SMS, email, websites, and social media—especially Facebook. A large majority of the respondents asked for more information regarding the environmental state of their community and the environmental hazards that may be related to their health in their everyday life (82% and 81%, respectively) (Figure 5). About half of the respondents reported using one or two sources to receive information about environmental hazards (47%), with TV being a main information source for 56% of the respondents, social media/internet for 54% of the respondents, and family/friends for 47% of the respondents (Figure 6). One stakeholder of the LAs mentioned:

*“If it is something immediate, we use the SMS. But there is Facebook, the website of the community; we use the mail for announcements. Also, if someone wants a meeting with me, there is personal phone and they can immediately visit me at the office or at home. There is direct personal contact with the residents, either in the cafes or in the office”*

(Interview 1, LA).

### 4.8. Usage of Exposome Concept by the Stakeholders

The exposome framework and its tools was not applied in the everyday or research and development (R&D) activities of SMIs, SMEs, and APA. However, the three stakeholder groups appeared willing to apply the concept and methodology of the exposome in the hydrocarbon industry with emphasis on occupational health.

*“The exposome could be used for evaluation. If your institute offers educational programs, we would like to attend. We want it to be part of our job because it complements us. We need such scientifically validated tools to help us communicate reliable information to the public”*

(Interview 1, SME).

Currently, SMEs and APA implement specific exposure measurements and measurements of air pollutants and volatile organic compounds in their outdoor and indoor activities, respectively. Moreover, as APA reported, there is a need for an overall health impact study on VEC and industrial facilities and, as mentioned above, the application of the exposome could be useful for this issue. The public, LA and governmental representatives were not asked about the exposome framework, which is still mostly research oriented and less applied in practice.

### 4.9. Compensatory Measures

The LA suggested that compensatory measures could help improve of the VEC area, such as tree planting, reforestation, financial support, and the construction of a community hospital. The public added that beach cleaning and the use of reusable energy could take place in the area, with one resident mentioning:

*“I am a young resident of the Psematismenos community. I wish to breathe clean oxygen for me and my children. We are suffering enough already from the heavy industries of the area. There is no place for more industrial development in the area, only for ecological development such as tree planting, beach cleaning, and reusable sources of energy”*

(Resident 49).

On the other hand, one stakeholder who represented local authorities added that:

*“[…] It is a financial issue and we fought a lot. Fortunately, the compensatory laws have been approved, of which 1.5 million will be given annually to the communities of Vasilikos, which means that each community will be able to offer a better quality of life […]”*

(Interview 8, LA).

Interviews, workshops, meetings, and questionnaires with the stakeholders of VEC paved the way for facilitating interactions and collaborations between the various stakeholders involved with the VEC industrial area for oil/gas strategic development of Cyprus. This investment for the future generations should abide by EU regulations on safety and health, ensuring the sustainable development of oil/gas for the next generations.

## 5. Discussion

A series of interviews, workshops, meetings, and questionnaires with the stakeholders of VEC area took place during 2019–2021 using a number of initiatives. The VEC hub development should abide by EU regulations on safety, health, and environmental aspects, ensuring the sustainable development of hydrocarbons in the region for the next generations. As such, a set of key messages, needs, and gaps emerged from the systematic engagement with all relevant stakeholders in the VEC, ranging from the government, the private sector, the research community, academia, local authorities, and the surrounding communities’ general populations. We synthesized the results of both the thematic analysis and the population survey and have presented the common points and differences that emerged through this stakeholder analysis.

According to the results of the current assessment, communities reported important safety and health risks associated with the establishment of the heavy industrial facilities in VEC. The SMEs reported the lack of systematic measurements on gaseous pollutant emissions in hydrocarbon activities in the surrounding communities of VEC. In addition, the rest of the involved stakeholders shared the desire and the need for an environmental and public health monitoring system in the VEC and surroundings via a joint effort by the government, private sector, and communities [11]. Moreover, the SMIs stated that there is no elevated risk to employees’ health, and that this is covered by companies′ health protocols that guarantee their employees′ physical and mental wellbeing. Furthermore, these stakeholders agreed that completing risk assessments and guaranteeing the health of employees and the general public in the VEC have both technical and financial challenges.

Despite the above community concerns, the environmental issues surrounding the communities of Vasilikos cannot be fully addressed without the cooperation of both the established industries and the government. Previous studies have shown the mistrust of government agencies and industries [2,4,31] and the need for collaborative knowledge between citizens, experts, scientists, and policy makers on equal terms [8]. In this study, the public blamed both community authorities for wrong decisions and partial flow of information, and the government for the initiative to transform the area into an industrial hub center. The SMEs, APA, and the government agreed that more risk communication to the public is necessary, proposing the implementation of public meetings and seminars to reduce misinformation and misunderstandings. Furthermore, SMEs and APA stated that governmental agencies have insufficient understanding and competence on hydrocarbon concerns. Alternatively, the government′s replies demonstrated a lack of expertise in managing health concerns, citing either that their department is not responsible to act/know or that other governmental agencies are responsible to act.

The societal decisions should be taken to balance acceptable levels of risk with expenditures for pollution prevention and control, water supply development and protection, and remediation [32,33]. The LA, SMEs, APA, and the general public agreed that the government needs to implement stricter policies and health monitoring actions. Moreover, it is noted that a large group of the community leaders consider community consultation to be a measure to protect public health and the quality of life of the population. This narrative agrees with many previous studies, which coined the term “risk communication”, on the effectiveness of this technique to increase the awareness of the public on environmental hazards [2,4,33,34,35].

The strengths of this study were the relatively good representation of all stakeholder groups with esteemed technical officers. The breadth of the stakeholder groups engaged allowed for the inclusion of all concerns and recommendations for the sustainable development of the VEC area. The fact that the general public was a key stakeholder in all these activities helped significantly to gain trust and enhance communication among stakeholders, providing a win-win situation for involved parties. However, the results presented here should be interpreted with caution because of limitations in the study design (i.e., the semi-structured interviews), including the small sample size (*n* = 309) and possible sampling bias, especially in the community survey, and the different methodologies used for different stakeholder groups. 

## 6. Conclusions

This exercise in stakeholder engagement on the health risks associated with the hydrocarbon developments in Cyprus’ main energy hub (VEC) highlighted a set of key messages, as well as gaps and needs in the form of recommendations for future steps. Almost all stakeholders expressed the immediate need for the design and execution of population health monitoring and impact studies for the VEC and its surrounding residential communities, including systematic measurements of gaseous pollutant emissions. Moreover, stricter policies by the government about licensing and monitoring of hydrocarbon activities and proper information dissemination to the public and the mass media emerged as important needs. Finally, usage of exposome concept was not practiced by the industry/SMEs, but SMIs in particular showed willingness to use it in the future, upon the proper testing/evaluation of its utility.

This 2-year-long stakeholder engagement process in the VEC and its surrounding area is considered vital to the health risk assessment process [35] and to the design and deployment of a public health monitoring system for the affected area. Consequently, the results and the findings of the current exercise were distributed back to the participated stakeholders for a complete evaluation feedback loop. Comments received were positive, and stakeholders unanimously supported activities to set up a systematic health monitoring system in the broader area surrounding the VEC. With regards to the human exposome concept and its use by the stakeholders, all considered it interesting and asked to learn more about it in the near future. The next steps of this engagement process include, but are not limited to, the organization of necessary actions to implement the mutually agreed recommendations and address major needs, which are the implementation of a health impact study (assessment of environmental and health risks in the VEC) and the exploration of future synergies and interactions in the field.

## Data Availability

The information and the data presented in this study is available in the Supplementary material.

## Supplementary Materials

The following are available online at https://www.mdpi.com/article/10.3390/ijerph182413133/s1, S1: Interview questions for local authorities of Vasilikos area, S2: Questionnaire for Vasilikos area residents, S3: Thematic Analysis’ Results, S4: Tables and Results of the Population Survey.

## References

[1] Byrd T., VanDerslice J., Peterson S. (1997). Variation in Environmental Risk Perceptions and Information Sources among Three Communities in El Paso. RISK Heal. Saf. Environ..

[2] Shin M., Werner A.K., Strosnider H., Hines L.B., Balluz L., Yip F.Y. (2019). Public Perceptions of Environmental Public Health Risks in the United States. Int. J. Environ. Res. Public Health.

[3] Baxter J., Lee D. (2004). Understanding Expressed Low Concern and Latent Concern near a Hazardous Waste Treatment Facility. J. Risk Res..

[4] Byrd T., VanDerslice J., Peterson S.K. (2001). Attitudes and Beliefs about Environmental Hazards in Three Diverse Communities in Texas on the Border with Mexico. Rev. Panam. Salud Publica/Pan Am. J. Public Health.

[5] Howel D., Moffatt S., Bush J., Dunn C.E., Prince H. (2003). Public Views on the Links between Air Pollution and Health in Northeast England. Environ. Res..

[6] Rahardyan B., Matsuto T., Kakuta Y., Tanaka N. (2004). Resident’s Concerns and Attitudes towards Solid Waste Management Facilities. Waste Manag..

[7] Rajapaksa D., Islam M., Managi S. (2018). Pro-Environmental Behavior: The Role of Public Perception in Infrastructure and the Social Factors for Sustainable Development. Sustainability.

[8] Uhl M., Santos R.R., Costa J., Santos O., Virgolino A., Evans D.S., Murray C., Mulcahy M., Ubong D., Sepai O. (2021). Chemical Exposure: European Citizens’ Perspectives, Trust, and Concerns on Human Biomonitoring Initiatives, Information Needs, and Scientific Results. Int. J. Environ. Res. Public Health.

[9] Venables D., Pidgeon N.F., Parkhill K.A., Henwood K.L., Simmons P. (2012). Living with Nuclear Power: Sense of Place, Proximity, and Risk Perceptions in Local Host Communities. J. Environ. Psychol..

[10] Ambituuni A., Amezaga J., Emeseh E. (2014). Analysis of Safety and Environmental Regulations for Downstream Petroleum Industry Operations in Nigeria: Problems and Prospects. Environ. Dev..

[11] Sam K., Coulon F., Prpich G. (2017). Use of Stakeholder Engagement to Support Policy Transfer: A Case of Contaminated Land Management in Nigeria. Environ. Dev..

[12] Haddad N., Andrianou X.D., Makris K.C. (2019). A Scoping Review on the Characteristics of Human Exposome Studies. Curr. Pollut. Rep..

[13] Wild C.P. (2005). Complementing the Genome with an “Exposome”: The Outstanding Challenge of Environmental Exposure Measurement in Molecular Epidemiology. Cancer Epidemiol. Biomark. Prev..

[14] Miller G.W., Jones D.P. (2014). The Nature of Nurture: Refining the Definition of the Exposome. Toxicol. Sci..

[15] Cyprus Department of Environment (2021). Στρατηγικη Μελετη Εκτιμησης Επιπτωσεων για το Χωροταξικο Σχεδιο Aναπτυξης της Περιοχης Βασιλικου.

[16] Noble Energy International (2015). Master Plan of the Vasilikos Area (Update) Executive Summary.

[17] Konstantinou C., Andrianou X.D., Constantinou A., Perikkou A., Markidou E., Christophi C.A., Makris K.C. (2021). Exposome Changes in Primary School Children Following the Wide Population Non-Pharmacological Interventions Implemented Due to COVID-19 in Cyprus: A National Survey. EClinicalMedicine.

[18] Andrianou X.D., Van Der Lek C., Charisiadis P., Ioannou S., Fotopoulou K.N., Papapanagiotou Z., Botsaris G., Beumer C., Makris K.C. (2019). Application of the Urban Exposome Framework Using Drinking Water and Quality of Life Indicators: A Proof-of-Concept Study in Limassol, Cyprus. PeerJ.

[19] European Commission (2015). Survey on Public Perceptions of Environmental Risks: Final Report.

[20] Rojas P., Neutra R. (2008). Stakeholder and Participant Involvement. Environmental Epidemiology: Study, Methods and Application.

[21] EURO-URHIS (2012). European Urban. Health Indications System Part 2.

[22] Republic of Cyprus Statistical Office (2016). European Health Survey 2014.

[23] Smyth C. (2000). The Pittsburgh Sleep Quality Index (PSQI). Director.

[24] Mason J. (2002). Qualitative Researching.

[25] Tong A., Sainsbury P., Craig J. (2007). Consolidated Criteria for Reporting Qualitative Research (COREQ): A 32-Item Checklist for Interviews and Focus Groups. Int. J. Qual. Heal. Care.

[26] Cassell C., Buehring A., Symon G., Johnson P., Bishop V. (2016). Qualitative Management Research: A Thematic Analysis of Interviews with Stakeholders in the Field.

[27] Merriam S.B. (2009). Transient Electromagnetic Topology Method for Complex. Wiring Consisting of Random and Nonuniform Transmission Lines.

[28] Braun V., Clarke V. (2006). Using Thematic Analysis in Psychology. Qual. Res. Psychol..

[29] R Core Team (2017). R: A Language and Environment for Statistical Computing.

[30] RStudio Team (1993). RStudio: Integrated Development Environment for R. Risk Anal..

[31] Canter L.W., Nelson D.I., Everett J.W. (2005). Public Perception of Water Quality Risks-Influencing Factors and Enhancement Opportunities. J. Environ. Syst..

[32] Levêque J.G., Burns R.C. (2019). Water Quality Perceptions and Natural Resources Extraction: A Matter of Geography?. J. Environ. Manag..

[33] Petts J. (2009). Handbook of Environmental Impact Assessment: Volume 2: Impact and Limitations.

[34] Steelman T.A., McCaffrey S. (2013). Best Practices in Risk and Crisis Communication: Implications for Natural Hazards Management. Nat. Hazards.

[35] Turner L.R., Alderman K., Connell D., Tong S. (2013). Motivators and Barriers to Incorporating Climate Change-Related Health Risks in Environmental Health Impact Assessment. Int. J. Environ. Res. Public Health.

