# Supplementary material for: Stakeholders′ Perceptions of Environmental and Public Health Risks Associated with Hydrocarbon Activities in and around the Vasilikos Energy Center, Cyprus
Source: Int J Environ Res Public Health. 2021 Dec 13;18(24):13133. doi: 10.3390/ijerph182413133 (PMC8702061; doi:10.3390/ijerph182413133)
Supplement: Supplementary file 1 [file ijerph-18-13133-s001.zip › ijerph-1478642-supplementary.pdf]

*Article*

# Stakeholders′ Perceptions of Environmental and Public Health Risks Associated with Hydrocarbon Activities in and around the Vasilikos Energy Center, Cyprus

**Eleni G. Kleovoulou [1], Corina Konstantinou [1], Andria Constantinou [1], Eelco Kuijpers [2], Miranda Loh [3], Karen S. Galea [3], Rob Stierum [2], Anjoeka Pronk [2] and Konstantinos C. Makris [1,*]**

[1] Cyprus International Institute for Environmental and Public Health, Cyprus University of Technology, Limassol 3041, Cyprus; eg.kleovoulou@edu.cut.ac.cy (E.G.K.); corina.konstantinou@cut.ac.cy (C.K.); andriaconstantinou95@gmail.com (A.C.); konstantinos.makris@cut.ac.cy (K.C.M.)

[2] The Netherlands Organization for Applied Scientific Research TNO, 3584 CB Utrecht, The Netherlands; eelco.kuijpers@tno.nl (E.K.); rob.stierum@tno.nl (R.S.); anjoeka.pronk@tno.nl (A.P.)

[3] Institute of Occupational Medicine (IOM), Edinburgh EH14 4AP, UK; miranda.loh@iom-world.org (M.L.); karen.galea@iom-world.org (K.S.G.)

* Correspondence: konstantinos.makris@cut.ac.cy

## Supplementary Information 1 (S1)

## Interview questions for local authorities of Vasilikos area

Name: ____________________________________________________

Profession: ____________________________________________________

Educational background: ____________________________________________

Organisation: ____________________________________________________

Community/ Postal Code: _____________________________________________

1. Could you tell me more about your community: what are the trends (growth, population characteristics, etc.)?
2. What are the biggest environmental concerns that you deal with in your community? And why?
3. Are there any environmental concerns in your community which are in relation to industries or agriculture? *(for example: toxins or viruses)*
4. What do you think the residents of your community consider being the hierarchically greatest threats to the health of their community? And why;
5. Is your work related to public health*? How?
6. Have residents of your community ever contacted you expressing their concerns about the environment? And what are the most common issues they raise?
7. How do you get in touch with the residents in your community?
8. What are the biggest opportunities for improving the quality of life and public health in your community?
9. What do you consider to be the biggest challenges / obstacles to improving the quality of life and public health in your community?
10. Is there anything else you would like to add that was not asked?

**Thank you.**

**Public health: public health is concerned with protecting the health of entire populations. These populations can be as small as a local neighbourhood, or as big as an entire country or region of the world. Public health is the science of protecting and improving the health of families and communities through promotion of healthy lifestyles, research for disease and injury prevention and detection and control of infectious diseases.*

## Interview questions for Government

1. Do you know what the national plan is for developing and monitoring hydrocarbon operations in Cyprus?
2. In what ways do your Department/Unit/Organization and yourself get involved in hydrocarbons operations?
3. Is enough attention being given to the health risks and their implications of hydrocarbons industry?
4. Does the national regulatory plan cover health risks of workers or residents affected by the hydrocarbons activities?
5. What actions can you take to ensure the health and safety of workers and/or residents? Are there any barriers preventing such progress?
6. Are there any health risks associated with hydrocarbons operations that are not currently addressed?
7. Do you think that the general community should be informed and involved in the integration and development of hydrocarbons industry in Cyprus-If yes, how?

## Interview questions for Academia/Professional Associations

1. Do you think that the general community should be informed and involved in the integration and development of hydrocarbons industry in Cyprus-If yes, how?
2. Do you think that Cyprus has the capacity to handle the safety and health implications of such hydrocarbon operations?
3. How can you help increase the capacity? Any barriers?
4. Are there any health risks associated with hydrocarbons operations that are not currently addressed?
5. Is there value in prioritizing the use of the exposomic framework for monitoring a) workers? b) for nearby residents?
6. How can the exposomic framework be applied to the hydrocarbon industry in order the assess the multiple exposures faced by a) workers and b) nearby residents?

# Interview questions for Industry

1. Do you think that the general community should be informed and involved in the integration and development of hydrocarbons industry in Cyprus-If yes, how?
2. How is your company involved in health risk assessment? What can you do better? What areas do you need assistance with? What are your barriers?
3. How is the health status of workers in the hydrocarbon industry monitored and assessed to the various exposures that are associated with the hydrocarbon industry?
4. Are there any health risks associated with hydrocarbons operations that are not currently addressed?
5. Is priority being placed on using the exposomic framework to monitor the effects of the hydrocarbon industry on a) workers' health? b) on nearby residents' health?
6. Is there interest in developing the exposomic framework to your monitoring procedures? If not, why not? If yes, how are you planning on including the exposome in monitoring

# Supplementary Information 2 (S2)

## Questionnaire for Vasilikos area residents

This questionnaire was developed to investigate the perceptions of Vasilikos area residents regarding the environment and health and to evaluate relevant environmental exposures.

**Important information:**

The questionnaire does not require the completion of personal data.

The questionnaire can be completed by adults who live in the following communities for at least 1 year: **Asgata, Hoirokoitia, Kalavasos, Marie, Maroni, Pentakomo, Psematismenos, Tochni and Zygi.**

The average time to complete the questionnaire is 10 - 15 minutes.

The study is conducted by the Cyprus International Institute for Environmental and Public Health (CII) of the Cyprus University of Technology and has been approved by the National Bioethics Committee of Cyprus (ΕΕΒΚ ΕΠ 2020.01.147).

You can find more information about the CII activities and our research team, using the below links:

CII: http://www.cut.ac.cy/cii/

Water and Health Laboratory: web.cut.ac.cy/waterandhealth/

Facebook: facebook.com/waterandhealthlab.CII/

Twitter: @waterhealth_lab

If you have any questions about the study, you can email us at CIIeph@cut.ac.cy or call: 25002282.

*It is very important to complete the below, prior to completing the questionnaire*

Please complete (X) if you agree to participate in the study:

I have read the information above and I agree to complete the questionnaire.

Date: ___________________________

**Voluntary consent for future communication**

Do you give us your consent to contact you in the future to inform you about the results of the study and / or to participate in a new phase of this study?

Your participation in the second phase of the study will be voluntary and you may decline in the future, even if you answer yes now.

☐ Yes

☐ No

Phone number:______________________________________________

Email:______________________________________________

*Thank you!*

## Health Assessment

**In general, how would you rate the condition of your health?**

☐ Very good
☐ Good
☐ Moderate
☐ Bad
☐ Very bad
☐ I don't know / I'm not sure / I can't remember
☐ I don't want to answer

**In general, do you have any concerns related to your health during this period of your life?**

☐ Yes
☐ A little
☐ No

**During the last 12 months have you been diagnosed with any of the following diseases by a doctor? Mark the relevant field.**

| | Yes | No | I don't know | I don't want to answer |
|---|---|---|---|---|
| Asthma (including allergic asthma) | | | | |
| Chronic bronchitis, emphysema | | | | |
| High blood pressure - Hypertension | | | | |
| Cardiovascular diseases | | | | |
| Diseases related to the joints of the body | | | | |
| Diseases related to the waist or other chronic neck problems | | | | |

| | | | | |
|---|---|---|---|---|
| Diabetes (excluding gestational diabetes) | | | | |
| Allergies (eg dermatitis) | | | | |
| Liver disease (e.g. cirrhosis of the liver) | | | | |
| Cancer (breast, prostate, lung, leukemia, etc.) | | | | |
| Depression | | | | |
| Any other disease (please specify): ____________________________ | | | | |

## Sleep quality

**What time do you usually go to sleep at?**

e.g. if you go to sleep at 11pm please write down 23:00 or if you go to sleep at 2am please write down 02:00

__________

**How many minutes does it usually take for you to fall asleep at night?;**

_________

**At what time do you usually wake up in the morning?**

e.g. if you wake up at 6am please write down 06:00 or if you wake up at 1pm please write down 13:00

_________

**How many hours of actual sleep do you get every night?**

These hours may differ from the time you spend laying down.

_________

## Perceptions of environmental issues

**How do you perceive the control over your life?**

Choose only one option

- ☐ My life is controlled by me, I decide what I want to do and how to succeed in life.

- ☐ My life is controlled by me and my family/friends, I involve others in my decision-making process, but I make the final decision.
- ☐ My life is controlled mainly by my family and/or friends
- ☐ My life is mainly controlled by my family/friends and the government.
- ☐ My life is mainly controlled by the government

**From the below, whose well-being is more important to you?**

Choose only one option

- ☐ My community's
- ☐ My friends'
- ☐ My family's
- ☐ My own well-being

**Having in mind your community, how would you overly rate it as a place of residence?**

Choose only one option

- ☐ Very good
- ☐ Good
- ☐ Somewhat good
- ☐ Somewhat bad
- ☐ Bad
- ☐ Very bad

**Do you believe that you live in a community where air pollution poses a problem?**

- ☐ Yes
- ☐ No
- ☐ I don't know

**Do you believe that you live in a community where water pollution poses a problem?**

- ☐ Yes
- ☐ No
- ☐ I don't know

**Do you believe that you live in a community where water is sometimes not safe to drink?**

- ☐ Yes
- ☐ No
- ☐ I don't know

**Do you believe that you live in a community where many people have the same health issues, such as cancer, asthma or cardiovascular diseases?**

- Yes
- No
- I don't know

**Do you believe that you live in a community where many people have been exposed to toxic waste?**

- Yes
- No
- I don't know

**If you answered «yes» to one of the previous questions (11-15), how worried are you that this has harmed your own health?**

- I am very worried
- I am somewhat worried
- I am not too worried
- I am not worried
- I don't know

**Do you drink tap water at your place of residence?**

- Yes
- Yes, but only if it is filtered
- No

**What are your main concerns when considering tap water as drinking water?**

*Choose only one option*

- Chemicals (e.g. heavy metals)
- Microbes (e.g. bacteria)
- Taste
- None
- Other (please specify): ____________________

**Having in mind your place of residence, how much do you worry about the following environmental factors?**

| | I am very worried | I am a little bit worried | I am not worried | I don’t know |
|---|---|---|---|---|
| Air quality | | | | |
| Quality of drinking water | | | | |
| Chemical substances | | | | |
| Food safety | | | | |
| Waste / rubbish | | | | |
| Noise | | | | |
| Contagious diseases (e.g. flu, COVID-19, infection by the west Nile virus) | | | | |

**During the last 12 months, whilst IN YOUR HOME, have you been exposed to any of the following environmental factors? If yes, to what degree?**

| | I have been exposed to a large degree | I have been exposed to a small degree | I have not been exposed | I don’t know |
|---|---|---|---|---|
| Noise (traffic, airplanes, factories, neighbours, animals, restaurants / bars / clubs) | | | | |
| Air pollution (dust, fumes) | | | | |
| Bad smells (industrial, agricultural, waste) | | | | |
| Water pollution (germs or chemicals in drinking water) | | | | |
| Food contamination (germs or toxic substances) | | | | |

**During the last 12 months have you been exposed to any of the following environmental factors whilst OUTSIDE your home (at your workplace or in your free time)? If yes, to what degree?**

| | I have been exposed to a large degree | I have been exposed to a small degree | I have not been exposed | I don't know |
|---|---|---|---|---|
| Noise (traffic, airplanes, factories, neighbours, animals, restaurants / bars / clubs) | | | | |
| Air pollution (dust, fumes) | | | | |
| Bad smells (industrial, agricultural, waste) | | | | |
| Water pollution (germs or chemicals in drinking water) | | | | |
| Food contamination (germs or toxic substances) | | | | |

## Environmental hazards and health

**How significant do you think environmental factors (for example, environmental pollution, toxic waste, etc.) are in the development of diseases or in causing diseases?**

- ☐ Very significant
- ☐ Somewhat significant
- ☐ Not so significant
- ☐ Not significant
- ☐ I don't know

**How dangerous do you consider the following environmental factors towards health?**

| | Very dangerous | Somewhat dangerous | A little dangerous | Not dangerous | I don't know |
|---|---|---|---|---|---|
| Air pollution | | | | | |
| Water pollution | | | | | |
| Chemical substances | | | | | |
| Noise | | | | | |
| Bad smells | | | | | |

| | | | | | |
|---|---|---|---|---|---|
| Rubbish / waste | | | | | |
| Food contamination | | | | | |
| Pathogenic microorganisms | | | | | |

**When considering the diseases found in the below table, do you believe that environmental factors play a big part, a small part or no part in their incidence?**

| | Big part | Small part | No part | I don't know |
|---|---|---|---|---|
| Cancer (lung, thyroid, breast, brain, prostate, etc.) | | | | |
| Tumours / Malignancies in children (e.g. leukemia) | | | | |
| Childhood asthma | | | | |
| Respiratory problems and allergies | | | | |
| Obesity | | | | |
| Type II diabetes | | | | |
| Disease caused by the new coronavirus (COVID-19) | | | | |
| Cold and flu | | | | |
| Diseases (e.g. malaria) that are transmitted by transmitters (e.g. mosquitos) | | | | |
| Contagious diseases such as measles, tuberculosis, hepatitis | | | | |
| Depression | | | | |

**Do you know someone personally whose health has been negatively affected by environmental factors?**

- ☐ Yes

- ☐ No

## Information sources related to environmental issues

**Do you believe that you have enough information regarding the environmental state of your community?**

- ☐ Yes, I have enough information
- ☐ I would like to have more information
- ☐ I do not want to receive this information
- ☐ I don't know

**Do you believe that you have enough information regarding the environmental hazards that are related to your health in your everyday life?**

- ☐ Yes, I have enough information
- ☐ I would like to have more information
- ☐ I do not want to receive this information
- ☐ I don't know

**In which of the following ways / with which of the following means do you receive information regarding environmental hazards?**
(You may choose more than one option)

- ☐ Newspapers / Magazines
- ☐ Family and/or friends
- ☐ Radio
- ☐ Social media and/or the internet
- ☐ TV
- ☐ Doctors
- ☐ Politicians
- ☐ Printed materials (publications, brochures)
- ☐ Books
- ☐ Events (conferences, lectures)
- ☐ None
- ☐ Other (please specify): ______________________

## Physical activity and eating habits

**How often do you carry out exercise which includes 30 minutes of continuous movement (e.g. fast walking, jogging, aerobic exercise, cycling)?**

- ☐ Every day
- ☐ 4-6 times per week
- ☐ 2-3 times per week
- ☐ Once per week
- ☐ 2-3 times per month
- ☐ Sometimes during the year
- ☐ I don't exercise due to health issues
- ☐ I don't exercise due to mobility issues
- ☐ I never exercise

**How often do you eat breakfast (e.g. cereal with milk, sandwich etc.)?**

- ☐ Every day
- ☐ 4-6 times per week

- ☐ 2-3 times per week
- ☐ Once a week
- ☐ 2-3 times per month
- ☐ I don’t eat breakfast

**How often do you eat ready-made food (take out) (e.g. from a restaurant, a fast-food restaurant etc.)?**

- ☐ Every day
- ☐ 4-6 times per week
- ☐ 2-3 times per week
- ☐ Once a week
- ☐ 2-3 times per month
- ☐ I do not eat ready-made food (take out)

**How often do you eat fruit;**

- ☐ Every day
- ☐ 4-6 times per week
- ☐ 2-3 times per week
- ☐ Once a week
- ☐ 2-3 times per month
- ☐ Once a month
- ☐ Never
- ☐ I don’t know
- ☐ I don’t want to answer

**On average, how many servings of fruit do you eat per day? ____________________**

***Note:*** *One serving is equivalent to approximately: 1 apple, 1 pear, 1 peach, 1 orange, 2 tangerines, 1 slice of watermelon / melon, 7 small strawberries, 14 cherries, 2 plums, 2 kiwis, 3 tablespoons of fruit salad (without compost or sugar), 1 small glass (150ml) of fresh fruit juice*

**How often do you eat vegetables?**

- ☐ Every day
- ☐ 4-6 times per week
- ☐ 2-3 times per week
- ☐ Once a week
- ☐ 2-3 times per month
- ☐ Once a month
- ☐ Never
- ☐ I don’t know
- ☐ I don’t want to answer

**On average, how many servings of vegetables do you eat per day? ____________________**

***Note:*** *One serving corresponds to: 2 pieces of broccoli, 2 large pieces of cauliflower, ½ a cup of cooked vegetables (e.g. green beans, cauliflower), 1 medium tomato, 1 cucumber, 1 carrot, 1 cup of fresh vegetable salad, 1 small drink (150ml) of fresh vegetable juice*

**How often do you eat meat;**

- ☐ Every day
- ☐ 4-6 times per week

- ☐ 2-3 times per week
- ☐ Once a week
- ☐ 2-3 times per month
- ☐ Once a month
- ☐ Never
- ☐ I don't know
- ☐ I don't want to answer

**On average, how many servings of meat do you eat per day?** ____________________
***Note:*** *One serving corresponds to: 90 grams of meat (pork, chicken, beef, lamb, goat, souvla, minced meat, souvlaki, kleftiko, pork chop, liver), 90 grams of lountza / ham, 1 slice of salami / bacon / mortadella, 120 grams of canned meat (e.g. ZWAN)*

**How often do you eat sugar (e.g. jam, honey, chocolate, biscuits, ice cream, cake)?**
- ☐ Every day
- ☐ 4-6 times per week
- ☐ 2-3 times per week
- ☐ Once a week
- ☐ 2-3 times per month
- ☐ Once a month
- ☐ Never
- ☐ I don't know
- ☐ I don't want to answer

**On average, how many servings of sugar do you eat per day?** ____________________
***Note:*** *One serving corresponds to: 1 teaspoon sugar / honey / jam / nutella, 45 grams of chocolate, 1 cookie, 1 ice cream ball, 1 piece of cake / tart, 1 bowl of pudding (e.g. rice pudding)*

## Smoking and alcohol consumption

**How often do you consume alcoholic beverages (of any kind)? (One portion corresponds to: 1 glass of beer / wine, 1 shot of brandy / zivania / whiskey / ouzo / liqueur, 1 alcoholic cocktail, 1 breezer)?**
- ☐ Daily or almost daily
- ☐ 5-6 times per week
- ☐ 3-4 times per week
- ☐ 1-2 times per week
- ☐ 2-3 times per month
- ☐ Once a month
- ☐ Less than once a month
- ☐ Never
- ☐ I don't know / I'm not sure / I don't remember
- ☐ I don't want to answer

**Do you smoke;**
- ☐ Yes, every day (*Proceed to questions 41.1 and 41.2*)
- ☐ Yes, occasionally (*Proceed to questions 41.1 and 41.3*)
- ☐ No *(Proceed to question 42)*
- ☐ I don't know / I'm not sure / I don't remember *(Proceed to question 42)*
- ☐ I don't want to answer

**If you answered «Yes» to the previous question**

**41.1 What type of products do you smoke?**

- ☐ Cigarettes (ready-made or roll ups)
- ☐ Cigars
- ☐ Pipe
- ☐ Electronic cigarette
- ☐ I don't know / I'm not sure / I can't remember

**If you answered «Yes, every day» to the previous question**

**41.2 How many cigarettes do you smoke during the day;**

- ☐ Number of cigarettes _______________
- ☐ I don't know / I'm not sure / I can't remember
- ☐ I don't want to answer

**If you answered «Yes, occasionally» to the previous question**

**How many cigarettes do you smoke during the week?**

- ☐ Number of cigarettes _______________
- ☐ I don't know / I'm not sure / I can't remember
- ☐ I don't want to answer

**How often are you exposed to second-hand smoke in closed areas, e.g. at home, at work, in public areas, in restaurants and whilst using public transport?**

- ☐ Never or almost never
- ☐ Less than 1 hour per day
- ☐ More than one hour per day
- ☐ I don't know / I'm not sure / I can't remember
- ☐ I don't want to answer
- ☐ Exposure to environmental factors and sensitivity

**Do you feel sick when exposed to smoke or specific smells, engine fumes (e.g. from vehicles), petrol fumes, air fresheners, pesticides? By sick we mean one of the following: headache, difficulty breathing, weakness, dizziness, upset stomach etc.**

Yes No

Please specify the product that negatively affects you: ________________________________________

**Are you unable to tolerate or do you have any unwanted/allergic reactions to medicines (antibiotics, analgesics, contraceptives) or other medical / surgical / dental materials or procedures?**

Yes No

**Are you unable to tolerate or do you have any unwanted/allergic reactions to food such as dairy products, wheat, corn, caffeine, eggs, alcoholic drinks or food colorings?**

Yes No

## Demographic data

**Sex:**

- ☐ Male
- ☐ Female
- ☐ I don't want to answer

**What is your year of birth?**

________

**What is your professional status?**

- ☐ Full time
- ☐ Part time
- ☐ Retired
- ☐ Non-profit
- ☐ Housework
- ☐ I don't work
- ☐ College/university Student
- ☐ Other (please specify):________

**What is your field of work?**

- ☐ Construction
- ☐ Agriculture / Fisheries
- ☐ Public sector
- ☐ Private sector
- ☐ Tourism
- ☐ Industrial (e.g. at a factory)
- ☐ Other (please specify):________

**What is your marital status?**

- ☐ Single
- ☐ Married (the cohabitation agreement is also included)
- ☐ Living with partner
- ☐ Widow / widower (not remarried)
- ☐ Divorced (not remarried)

**Do you own or rent the house you live in?**

- ☐ Owner
- ☐ Tenant
- ☐ Other ______________________

**What is the highest level of education you have successfully completed?**

- ☐ No education
- ☐ I have not completed primary school
- ☐ Primary school graduate
- ☐ High school graduate (3 classes)
- ☐ Lyceum, technical / professional school graduate
- ☐ Post-secondary non-tertiary education (<2 years)
- ☐ Tertiary non-university (college <4 years)
- ☐ Tertiary (3-4 years University, Bachelor's etc.)
- ☐ Master's degree

- ☐ PhD

**Where were you born:**

- ☐ Cyprus
- ☐ In another country of the European Union
- ☐ Please specify: ______________
- ☐ In a country outside the European Union
- ☐ Please specify: _________________

**Where is your current place of residence?**

- ☐ Mari
- ☐ Zigi
- ☐ Pentakomo
- ☐ Asgata
- ☐ Kalavasos
- ☐ Psematismenos
- ☐ Maroni
- ☐ Choirokoitia
- ☐ Tochni
- ☐ Other. Please specify: _________________________

**How long have you been living at your place of residence?**

- ☐ Less than 1 year
- ☐ 1 to 5 years
- ☐ 6 to 10 years
- ☐ 11 to 20 years
- ☐ Over 20 years

**Do you own or rent the house that you live in;**

- ☐ Owner
- ☐ Tenant
- ☐ Other (please specify): _____________

**How many children under the age of 18 live in your home?**

- ☐ 0
- ☐ 1
- ☐ 2
- ☐ 3
- ☐ 4
- ☐ 5 or more

**Height (without shoes)?**
In cm
________

**Weight (without shoes)?**
In kg
________

Thank you for your participation!

If you have any comments, you may write them below:

**Please place the completed questionnaire (s) in the envelope that we have provided. Seal the envelope and return it to your Community Council office!**

# Supplementary Information 3 (S3)

## Thematic Analysis' Results

| Synthesizing stakeholders' engagement process | | | | | | |
|---|---|---|---|---|---|---|
| **CODES** | **Local Authorities** | **Small Medium Industries** | **SMEs** | **Academia** | **Government** | **Public** |
| **Assessment of health and safety issues** | **Activities**<br>• Environmental measurements and air quality studies in collaboration with governmental and private institutes using communities' own income | **Activities**<br>• Onsite risk assessment<br>• Onsite noise and air measurements | **Activities**<br>• Risk assessments in each entity/organization/platform<br>• Air and water quality measurements<br>• Reduction of environmental pollutants in laboratories | **Not Applicable** | **Activities**<br>• Risk assessment in each entity/organization<br>• Risk assessment into industrial areas and the VEC<br>• Technical committee for Vasilikos area | **Not Applicable** |
| | **Needs**<br>• More environment and health studies<br>• Air and water quality measurements<br>• Collaboration with scientists | **Needs**<br>• More environment and health studies around such industries<br>• Measurements on gaseous pollutant emissions in hydrocarbon facilities | **Needs**<br>• Health impact study of VEC industrial facilities on surrounding populations<br>• Measurements on gaseous pollutant emissions in hydrocarbon facilities | **Needs**<br>• Health impact study of VEC industrial facilities on surrounding populations<br>• Specializing skills or field experience for addressing safety or health issues of hydrocarbons<br>• Train the trainer: Dissemination of knowledge by foreign experts<br>• Absence of specializing equipment in measuring hydrocarbons and biomarkers<br>• Development of curricula for hydrocarbons training | **Needs**<br>• Health impact study of VEC industrial facilities on surrounding populations<br>• Air quality measurements and emissions (e.g., BTEX)<br>• Pollution control measures in the Vasilikos area<br>• Epidemiological data will help in updating current law for gas stations | |

| | | | | | | |
|---|---|---|---|---|---|---|
| **Safety Risks in the hydrocarbons industry** | **Risks**<br>• Accumulation of industries at VEC<br>• Increased risk of an industrial accident<br>• Human mistake/sabotage – Comparison with Mari explosion | **Risk Avoidance**<br>• Implementation of safety protocols for infrastructure and employees at workplace<br>• Personal protective equipment<br>• Development and implementation of health and safety system<br>• High standards and implementation of legislation | **Risk Avoidance**<br>• High standards in every procedure<br>• Implementation of the EU Directive on industrial emissions<br>• Safety on-site inspections in infrastructure, land and sea<br>• Evaluation of results based on legislative provisions | **Risks**<br>• Accumulation of industries at VEC and lack of space in Cyprus<br>• Equipment and infrastructure at various levels not specialized enough<br>• Lack of EU-standardized safety protocols for equipment in hydrocarbons exploration<br>• Weak standards for processing and storage of oil/gas<br>• Amplification of the climate crisis (greenhouse effect) | **Risk Avoidance**<br>• SEVESO plan<br>• ZENON plan<br>• Onsite Inspections<br>• Hydrocarbons Regulations<br>• Protocol on the Protection of the Mediterranean Sea against Pollution<br>• VEC Master Plan | **Risks**<br>• Accumulation of industries at VEC<br>• Wrong handling and operating conditions in industries<br>• Uncertain future for the next generations |
| **Health Risks in the hydrocarbons industry** | **Risks**<br>• Increased disease cases burdens in the community (cancer, respiratory, heart, thyroid) | **Risk Avoidance**<br>• No emerging risk on employees' health<br>• No risks because of off-shore exploration status<br>• On-site noise and air measurements<br>• On-site risk assessment<br>• Annual medical check and psychological support to employees<br>• Public health is not affected (acceptable risks)<br>**Risks**<br>• Long-term exposure of employees by many hydrocarbons industries operating in a small area (VEC) | **Needs**<br>• Health impact study of VEC industrial facilities on surrounding populations | **Needs**<br>• Health impact study of VEC industrial facilities on surrounding populations<br>• Cooperation between governmental departments, companies and communities | **Needs**<br>• Health impact study of VEC industrial facilities on surrounding populations<br>• Cooperation between governmental departments, companies and communities<br>• Performance of a project, considering the proximity of residence from gas stations | **Risks**<br>• Cancer<br>• Asthma<br>• Heart diseases<br>• Thyroid diseases<br>• Prostate<br>• Thymus<br>• 42% being diagnosed with waist- or joint-related diseases or chronic neck problems<br>• 34% being diagnosed with hypertension<br>• 19% asthma/bronchitis/allergies<br>• 14% cardiovascular diseases |

| | | | | | | |
|---|---|---|---|---|---|---|
| | | • Accumulation of industries in the VEC<br><br>**Needs**<br>• Health-based risk assessment in the VEC<br>• Specialized protection equipment<br>• Epidemiological studies for populations around such facilities<br>• Cooperation between governmental departments, companies and communities<br>• Performance of a project, considering the proximity of residence from gas stations | | | | • 14% depression<br>• 13% diabetes<br>• 9% other diseases<br>• 7% cancer<br>• 1% liver disease |
| **Environmental risks** | **Risks**<br>• Pollutants<br>• Water pollution<br>• Dust<br>• Odors<br>• Noise<br>• Visual pollution<br>• Fear of contamination of drinking water due to industry<br>**Needs**<br>• Need for the industry to comply with environmental limits and standards<br>• Installation of air filters<br>• Negligible communication with industries | **Not Applicable** | **Needs**<br>• Absence of measurements on gaseous pollutant emissions in hydrocarbon facilities | **Not Applicable** | **Activities**<br>• Environmental Impact Assessment Committees<br>• Ad-hoc Committee on the Offshore Protocol<br>• Offshore Oil and Gas Technical Committee<br>• Safety, Health and Environment Monitoring Committee of the VEC<br>• Committee for the Review of the Spatial Development Plan of the Vasilikos area<br>• Committee on Strategic Planning for the Creation of a New | **Risks**<br>• Pollution from the use of gases, waste, chemicals, odors, dust, smoke and radiation<br>• Residents (%) believe that they live in a community with:<br>87% air pollution<br>81% toxic waste<br>70% water pollution<br>  • 39% reported that chemicals were their main concerns regarding tap water, while,<br>  • 26% considered microbes as the main concern |

| | | | | | | |
|---|---|---|---|---|---|---|
| | | | | | Industrial Port in the Vasilikos Area<br>• Evaluation Committee of the oil/gas Development and Exploitation Plan<br>• Cyprus Parliament | |
| **Sharing responsibility on health issues** | **Current situation**<br>• The government is not as strict as should be for the licensing process of a new industry in VEC<br>• Wrong government decisions<br>• Unwillingness of the government to help in finding a viable and functional solution | **Not Applicable** | **Current situation**<br>• Government should implement stricter policies and monitoring actions<br>• Incomplete knowledge and expertise from the government on hydrocarbon issues | **Current situation**<br>• Absence of risk monitoring by the government<br>• Incomplete knowledge and expertise from the governmental authorities on hydrocarbon issues<br>• Need to collaborate with the government | **Current situation**<br>• Their department is not responsible to know<br>• Other governmental departments are responsible<br>• Lack of knowledge in these issues | **Current situation**<br>• Community authorities take inappropriate decisions<br>• Government's false decisions to turn the Vasilikos area into an industrial hub |
| **Usage of Exposome concept** | **Not Applicable** | **No usage**<br>• Willing to use exposome concept in the future | **No usage**<br>• Currently: Specific exposure measurements<br>• Unknown concept<br>• Interest in the application of exposome concept | **No usage**<br>• Currently: Measurement of air pollutants and volatile organic compounds in open and closed environments<br>• Exposome utility on health impact study on VEC and industrial facilities<br>• Interest in application of the exposome concept | **Not Applicable** | **Not Applicable** |
| **Risk communication** | **Current actions**<br>• Uninterrupted physical communication with the residents | **Current actions**<br>• The obligation to inform the public should be respected | **Needs**<br>• Enhancing public consultations, | **Needs**<br>• Much needed information to the public because of the | **Needs**<br>• Need for evidence-based and transparent | **Needs**<br>• Lack of information by communities' authorities |

| | | | | | | |
|---|---|---|---|---|---|---|
| | • Communication through new media<br>• Community consultations<br>• Information printed material | • Communication with the public before licensing of a new industry for alleviating misinformation and concerns<br>• Public seminars and in – school information should take place<br>• Taking serious notes on public concerns and issues expressed to them<br>• Stakeholder engagement via the Vasilikos-government-industry technical committee | seminars and press releases<br>• Reduce misinformation by mass media and politicians | first time ever that the natural gas discussed in Cyprus<br>• Inform the public before the licensing of a new industry<br>• The obligation to inform the public should be respected<br>• Reduce misinformation by mass media<br>• Need for general public consultation, seminars and press releases<br>• Inclusion of experts during public consultation | information to public and mass media<br>• Need for more public consultation and seminars for the public | **Sources of information**<br>• 56% - TV<br>• 54% - Social media/internet<br>• 47% - Family/friends<br>• 35% - Newspapers/magazines<br>• 33% - Radio<br>• 27% - Events<br>• 20% - Printed material<br>• 20% - Doctors<br>• 10% - Books<br>• 4% - None<br>• 2% - Politicians<br>• 2% - Other |
| **Compensatory measures** | **Suggestions**<br>• Tree planting<br>• Reforestation<br>• Financial governmental support to improve the Vasilikos area<br>• Hospital establishment | **Not Applicable** | **Not Applicable** | **Not Applicable** | **Not Applicable** | **Suggestions**<br>• Tree planting<br>• Beach cleaning<br>• Use of reusable energy sources |

# Supplementary Information 4 (S4)

## Tables and Results of the Population Survey

# Tables – Figures

Table S1 **Demographics and other characteristics of respondents**

| | Overall | Male | Female |
|---|---|---|---|
| **N (%)** | 308 | 146 (49) | 154 (51) |
| **Age (mean (SD))** | 54.91 (17.61) | 56.70 (17.38) | 53.10 (17.74) |
| **Age groups (%)** | | | |
| 18-29 | 28 (10) | 10 (7) | 18 (12) |
| 30-44 | 70 (24) | 32 (23) | 37 (25) |
| 45-59 | 62 (21) | 28 (20) | 34 (23) |
| >59 | 134 (46) | 71 (50) | 61 (41) |
| **BMI (median [IQR])** | 26.23 [22.83, 29.60] | 27.68 [24.52, 30.04] | 24.03 [21.08, 28.30] |
| **BMI groups (%)** | | | |
| Underweight | 6 (2) | 0 (0) | 6 (4) |
| Normal weight | 111 (38) | 40 (29) | 71 (49) |
| Overweight | 106 (37) | 64 (46) | 40 (28) |
| Obese | 67 (23) | 36 (26) | 28 (19) |
| **Place of residence (%)** | | | |
| Mari | 8 (3) | 3 (2) | 5 (3) |
| Zygi | 33 (11) | 17 (12) | 15 (10) |
| Pentakomo | 71 (23) | 33 (23) | 37 (24) |
| Asgata | 17 (6) | 8 (5) | 9 (6) |
| Kalavasos | 80 (26) | 39 (27) | 38 (25) |
| Psematismenos | 62 (20) | 32 (22) | 29 (19) |
| Maroni | 19 (6) | 7 (5) | 11 (7) |
| Choirokoitia | 12 (4) | 5 (3) | 7 (5) |
| Tochni | 5 (2) | 2 (1) | 2 (1) |
| **Years living in community (%)** | | | |
| 1 to 5 years | 10 (3) | 7 (5) | 2 (1) |
| 6 to 10 years | 15 (5) | 10 (7) | 4 (3) |
| 11 to 20 years | 60 (20) | 28 (19) | 29 (19) |
| over 20 years | 220 (72) | 100 (69) | 117 (77) |

| | | | |
|---|---|---|---|
| **Place of birth (%)** | | | |
| Cyprus | 285 (93) | 139 (95) | 139 (90) |
| EU country | 16 (5) | 6 (4) | 10 (6) |
| Non-EU country | 6 (2) | 1 (1) | 5 (3) |
| **Household (%)** | | | |
| Owner | 251 (83) | 120 (83) | 126 (83) |
| Tenant | 25 (8) | 13 (9) | 11 (7) |
| Other | 27 (9) | 12 (8) | 15 (10) |
| **Education level (%)** | | | |
| Has never been to school | 7 (2) | 2 (1) | 4 ( 3) |
| Has not completed primary school | 11 (4) | 6 (4) | 5 ( 3) |
| Primary School | 70 (23) | 33 (23) | 34 (22) |
| Middle School (3 years) | 23 (8) | 8 (6) | 15 (10) |
| Lyceum, technical / professional school | 90 (30) | 50 (35) | 39 (25) |
| Post-secondary non-tertiary education (<2 years) | 13 (4) | 6 (4) | 7 (5) |
| Tertiary Education (non-Univesity) (college <4 years) | 19 (6) | 8 (6) | 11 (7) |
| University (Bachelor’s degree) | 36 (12) | 14 (10) | 22 (14) |
| University-Postgraduate (Master’s degree) | 30 (10) | 12 (9) | 17 (11) |
| PhD | 3 (1) | 2 (1) | 0 (0) |
| **Marital status (%)** | | | |
| Single | 34 (11) | 14 (10) | 19 (12) |
| Married | 237 (79) | 121 (85) | 112 (73) |
| Living with partner | 7 (2) | 2 (1) | 5 (3) |
| Widower and not remarried | 14 (5) | 1 (1) | 13 (8) |
| Divorced | 9 (3) | 4 (3) | 4 (3) |
| **No of children (%)** | | | |
| 0 | 175 (62) | 84 (62) | 86 (61) |
| 1 | 35 (12) | 15 (11) | 20 (14) |
| 2 | 50 (18) | 26 (19) | 23 (16) |
| 3 | 19 (7) | 8 (6) | 11 (8) |

| | | | |
|---|---|---|---|
| 4 | 2 (1) | 0 (0) | 2 (1) |
| 5 or more | 3 (1) | 2 (1) | 0 (0) |
| **Professional status (%)** | | | |
| Full-time | 121 (40) | 68 (47) | 51 (33) |
| Part-time | 23 (8) | 7 (5) | 16 (10) |
| Retired | 109 (36) | 60 (41) | 46 (30) |
| Non-profit | 1 (0) | 1 (1) | 0 (0) |
| Housework | 27 (9) | 1 (1) | 26 (17) |
| Unemployed | 10 (3) | 2 (1) | 8 (5) |
| College/University Student | 10 (3) | 3 (2) | 7 (5) |
| Other | 3 (1) | 3 (2) | 0 (0) |
| **Job sector (%)** | | | |
| Constructions | 9 (6) | 9 (12) | 0 (0) |
| Agriculture/Fisheries | 8 (6) | 5 (7) | 3 (4) |
| Public Sector | 32 (23) | 17 (23) | 14 (21) |
| Private Sector | 69 (49) | 27 (37) | 42 (63) |
| Tourism | 5 (4) | 2 (3) | 2 (3) |
| Industry | 12 (8) | 9 (12) | 3 (4) |
| Other | 7 (5) | 4 (5) | 3 (4) |

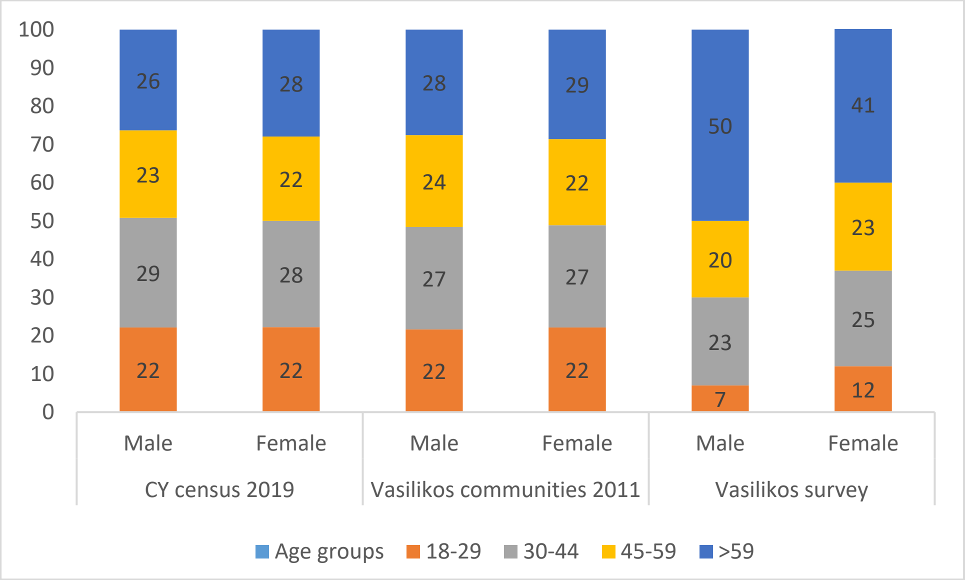


**Figure S1** Age distribution by sex in CY census 2019, Vasilikos communities 2011 and survey respondents.

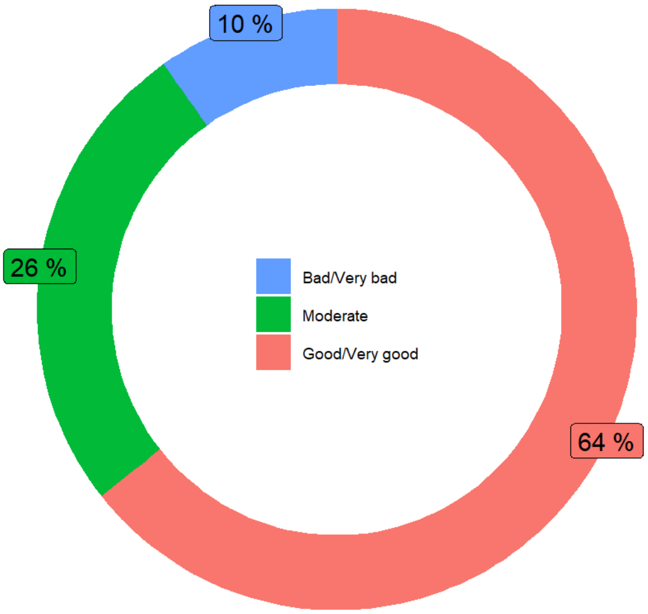


**Figure S2** Self-assessed health

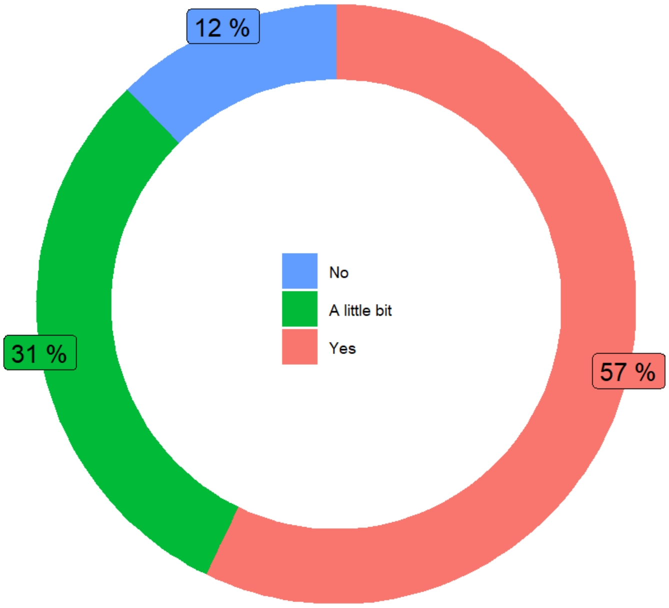


**Figure S3** Perceived worry about health (Question: In general, are you worried for your health during this period?)

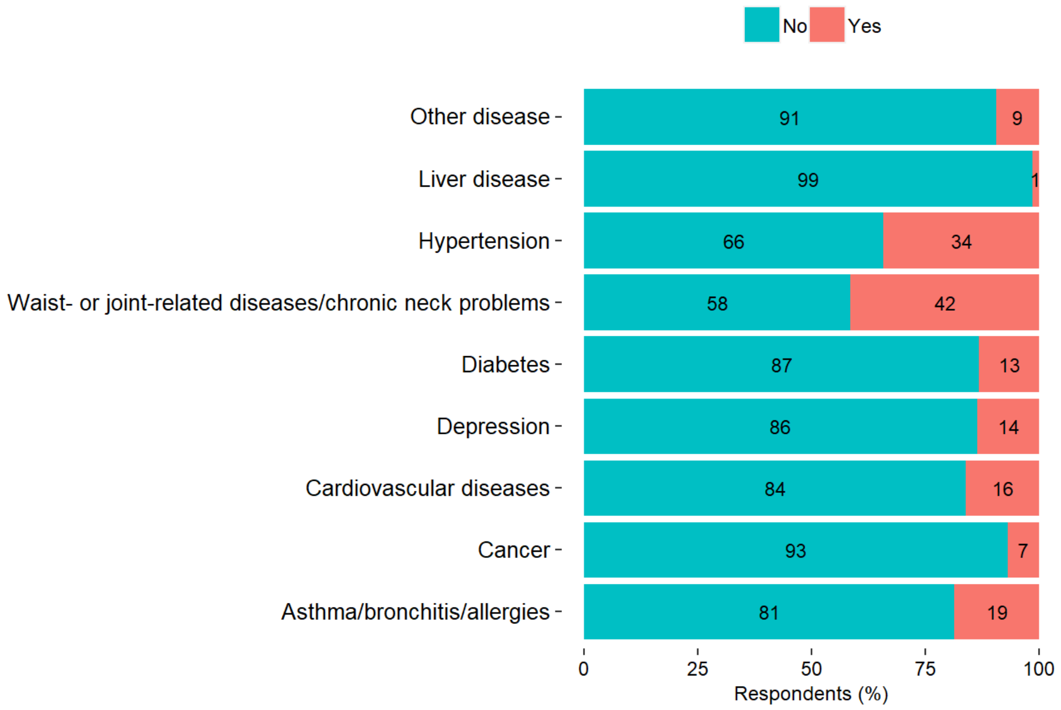


**Figure S4** Diseases during the last 12 months (Question: During the last 12 months have you been diagnosed with any of the following diseases by a doctor?)

**Table S2** Sleep quality parameters

| | **Overall** | **Males** | **Females** |
|---|---|---|---|
| n | 304 | 144 | 153 |
| **Minutes to sleep score (%)** | | | |
| 3 | 34 (12.3) | 13 (9.8) | 20 (14.2) |
| 2 | 85 (30.7) | 36 (27.3) | 47 (33.3) |
| 1 | 33 (11.9) | 21 (15.9) | 12 (8.5) |
| 0 | 125 (45.1) | 62 (47.0) | 62 (44.0) |
| **Sleep duration score (%)** | | | |
| 3 | 11 (5.3) | 4 (4.3) | 7 (6.4) |
| 2 | 28 (13.6) | 13 (14.0) | 13 (11.9) |
| 1 | 52 (25.2) | 22 (23.7) | 30 (27.5) |
| 0 | 115 (55.8) | 54 (58.1) | 59 (54.1) |
| **Habitual sleep efficiency score (%)** | | | |
| 3 | 14 (7.6) | 6 (7.5) | 8 (8.0) |
| 2 | 17 (9.2) | 4 (5.0) | 11 (11.0) |

| | | | |
|---|---|---|---|
| 1 | 32 (17.4) | 14 (17.5) | 17 (17.0) |
| 0 | 121 (65.8) | 56 (70.0) | 64 (64.0) |

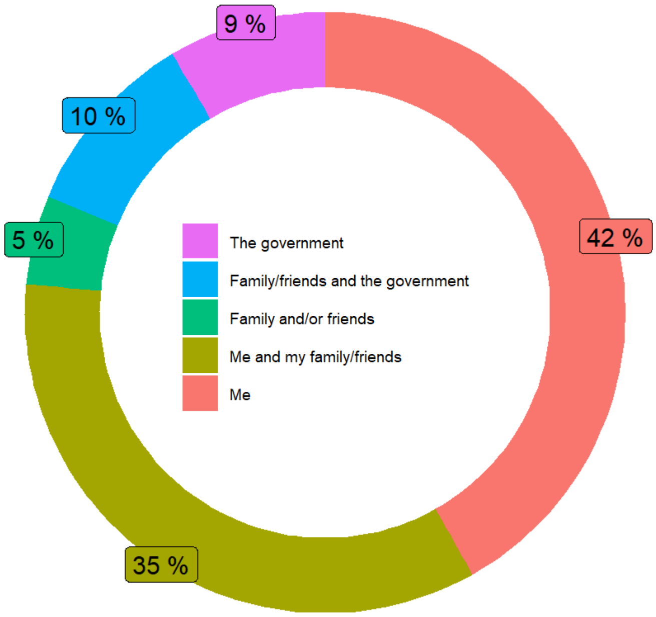


**Figure S5** Perceived control over life (Question: How do you perceive the control over your life?)

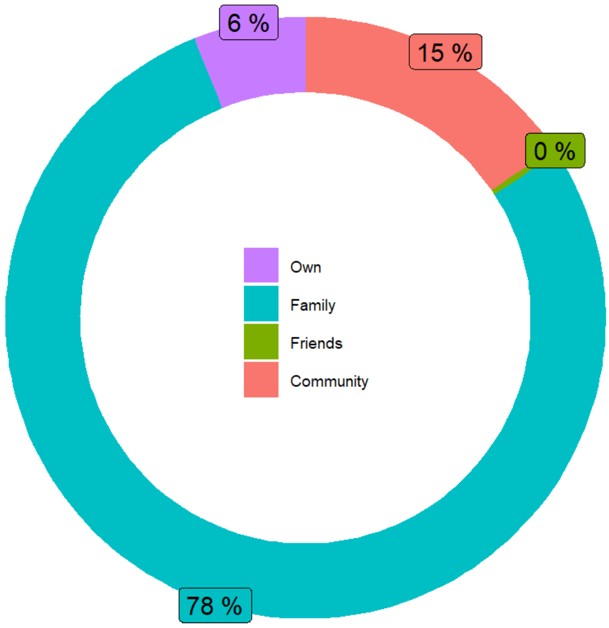


**Figure S6** Well-being importance (Question: Whose well-being is more important to you?)

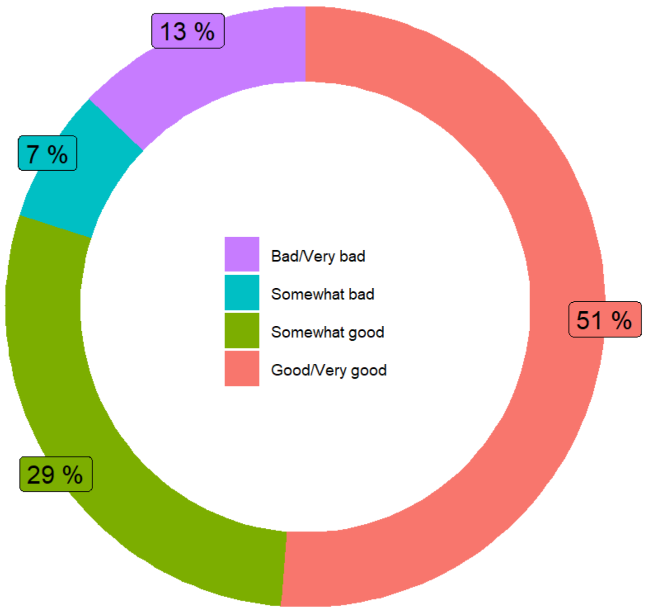


**Figure S7** Rate of community as place of residence (Question: Having in mind your community, how would you overly rate it as a place of residence?)

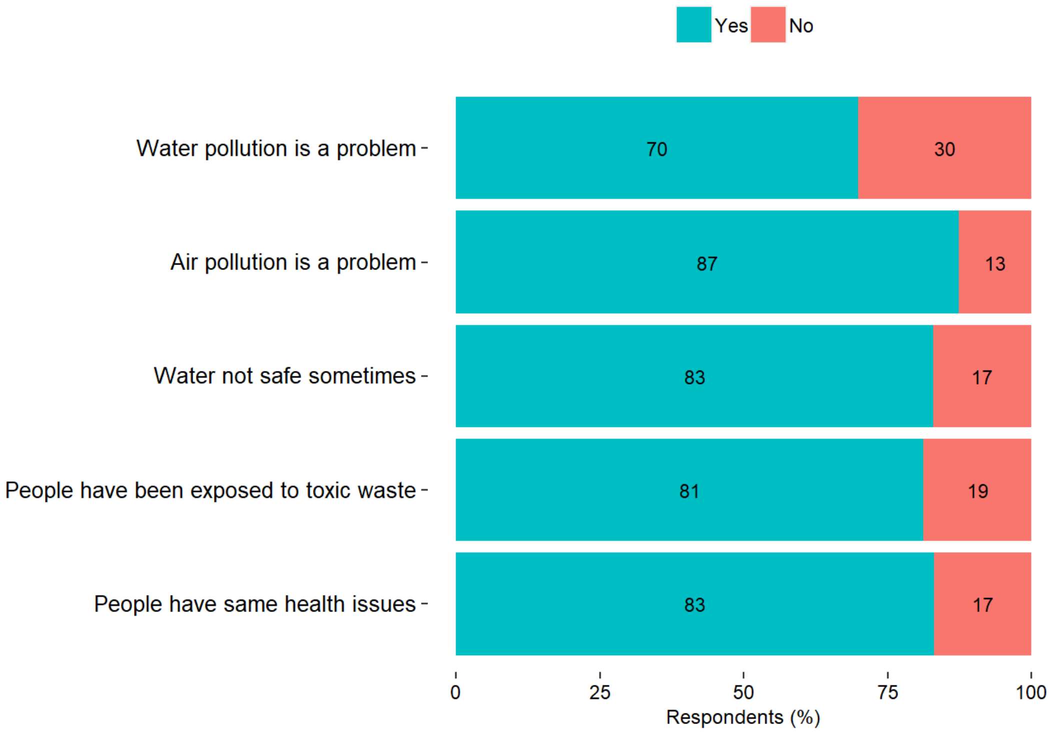


**Figure S8** Perceptions about community issues (Do you believe that you live in a community where: (i) air pollution poses a problem? (ii) water pollution poses a problem? (iii) water is not safe sometimes? (iv) many people have the same health issues such as cancer, asthma, CVDs? (v) many people have been exposed to toxic waste?)

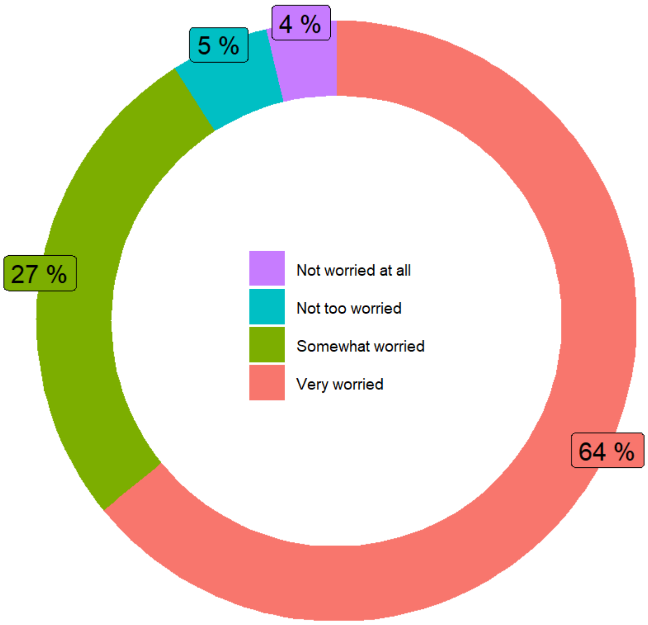


**Figure S9** Perceived level of worry about issues in community (mentioned in figure 8) (Question: If you responded "Yes" to the previous questions (11-15), how much do you worry that this has harmed your health?)

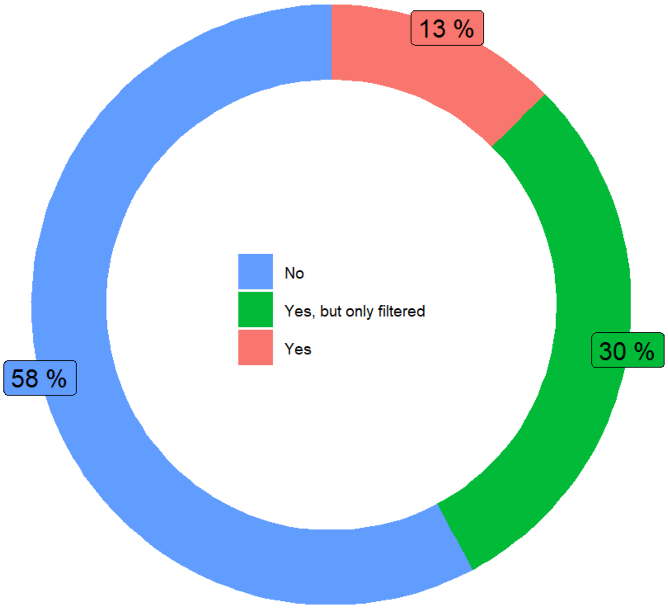


**Figure S10** Drinking of tap water at place of residence (Question: Do you drink tap water at your place of residence?)

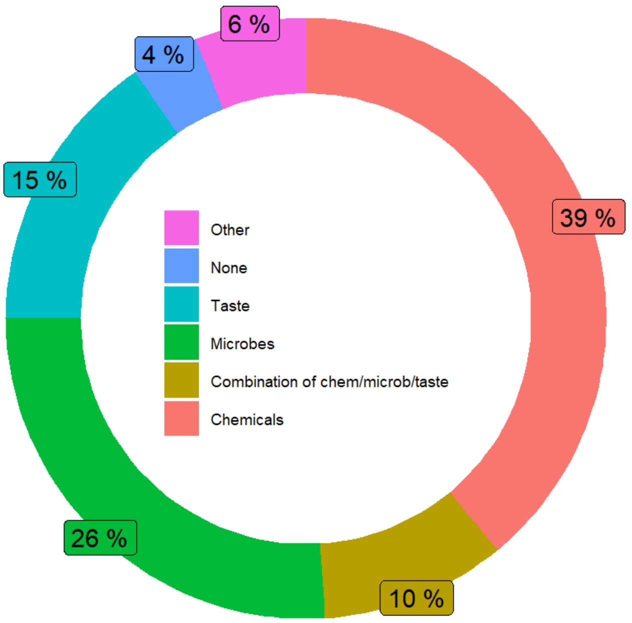


**Figure S11** Concerns for tap water as drinking water (Question: What are your main concerns when considering tap water as drinking water?)

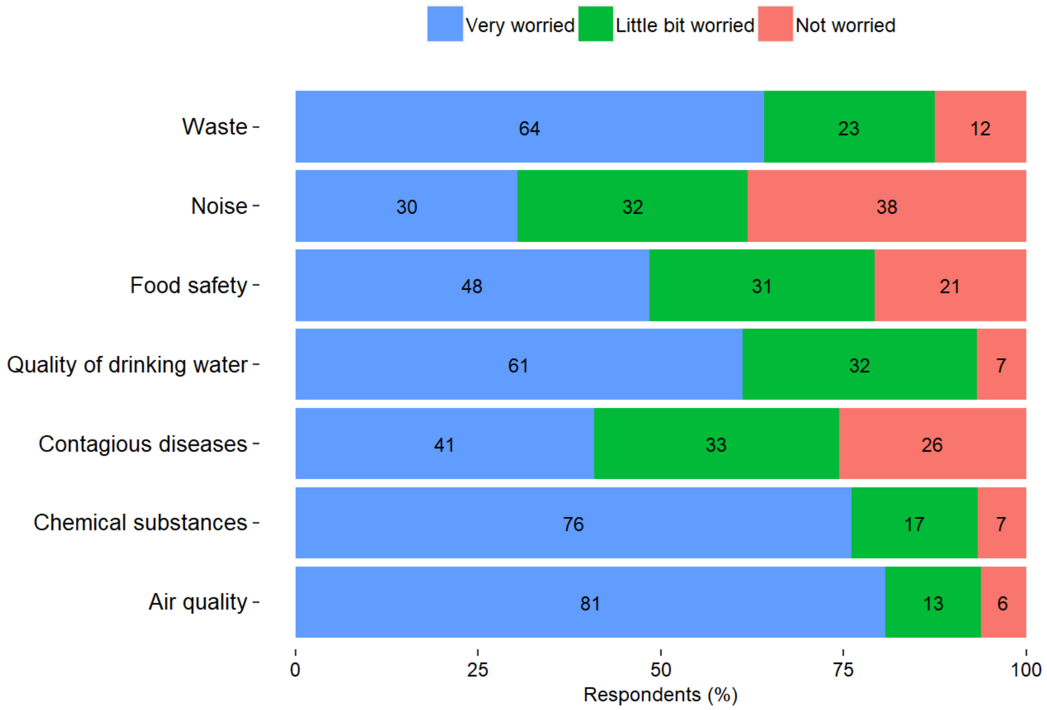


**Figure S12** Perceived level of worry for environmental factors in the place of residence (Question: Having in mind your place of residence, how much do you worry about the following environmental factors?)

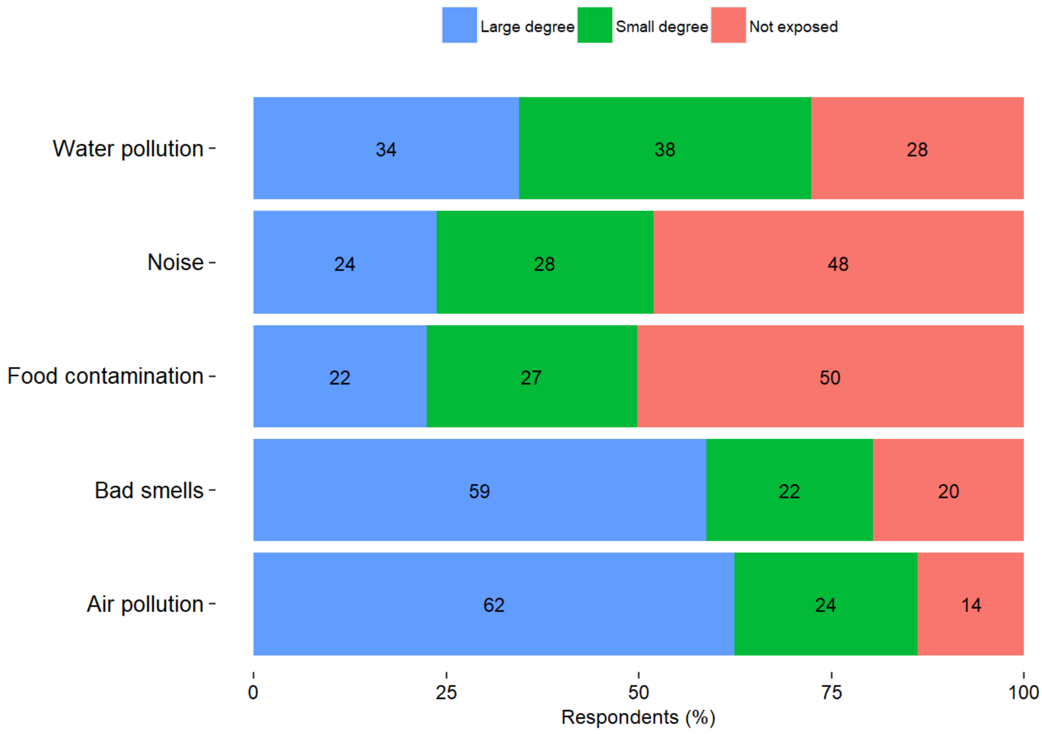


**Figure S13** Perceived degree of exposure in environmental factors, whilst at home, during the past 12 months (Question: During the last 12 months, whilst IN YOUR HOME, have you been exposed to any of the following environmental factors?)

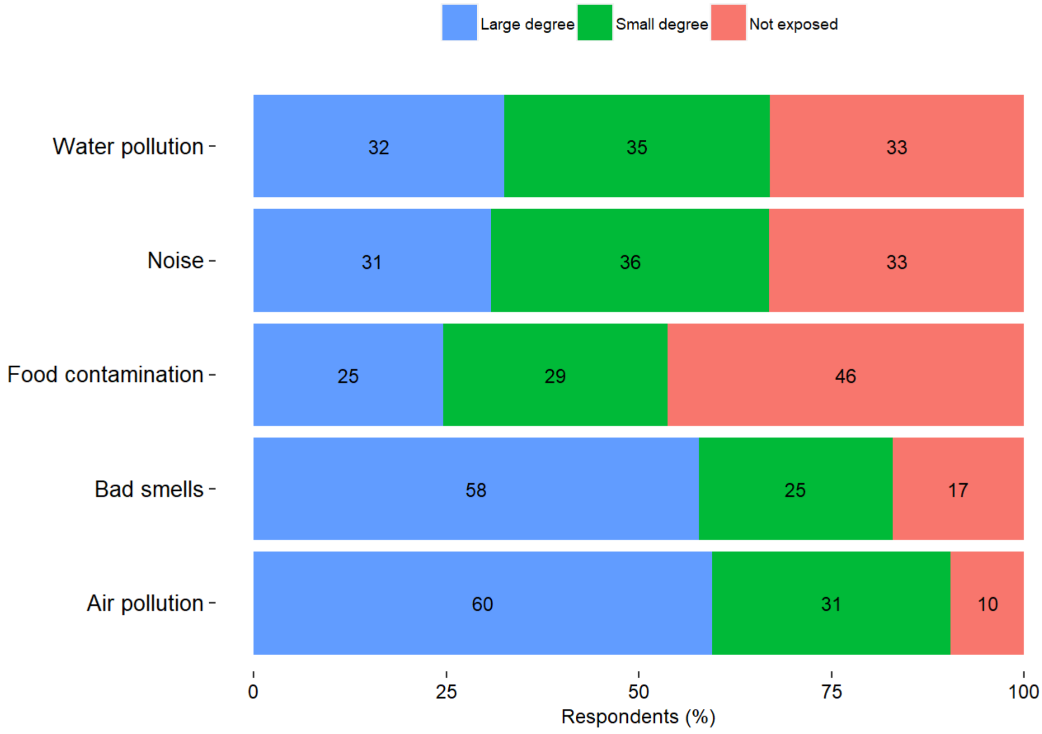


**Figure S14** Perceived degree of exposure in environmental factors, whilst outside their home, during the past 12 months (Question: During the last 12 months, whilst OUTSIDE YOUR HOME, have you been exposed to any of the following environmental factors?)

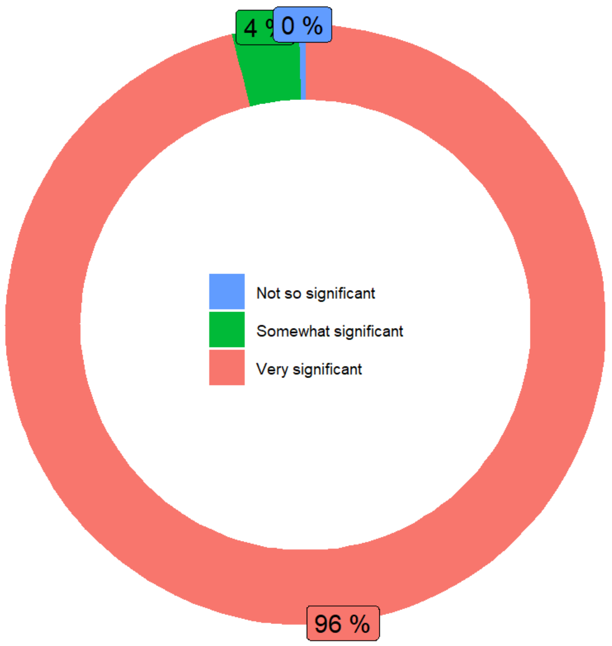


**Figure S15** Perceived significance of environmental factors in disease cause or development (Question: How significant do you think environmental factors are in causing or in the development of diseases?)

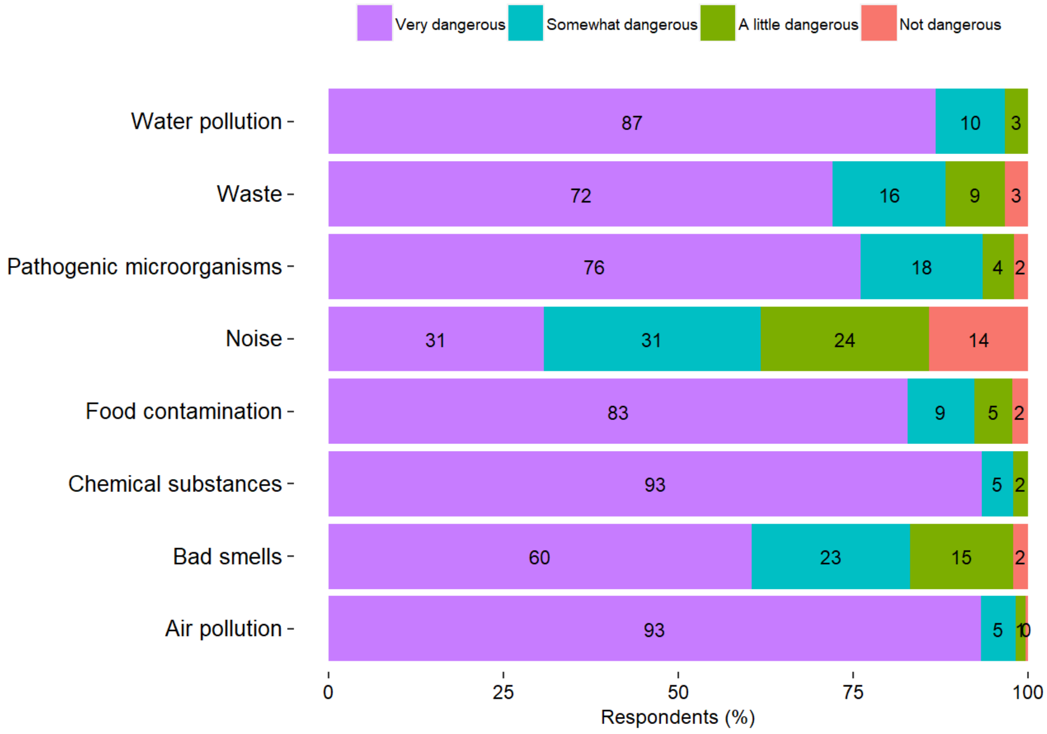


**Figure S16** Perceived danger level of environmental factors for health (Question: How dangerous do you consider the following environmental factors for health?)

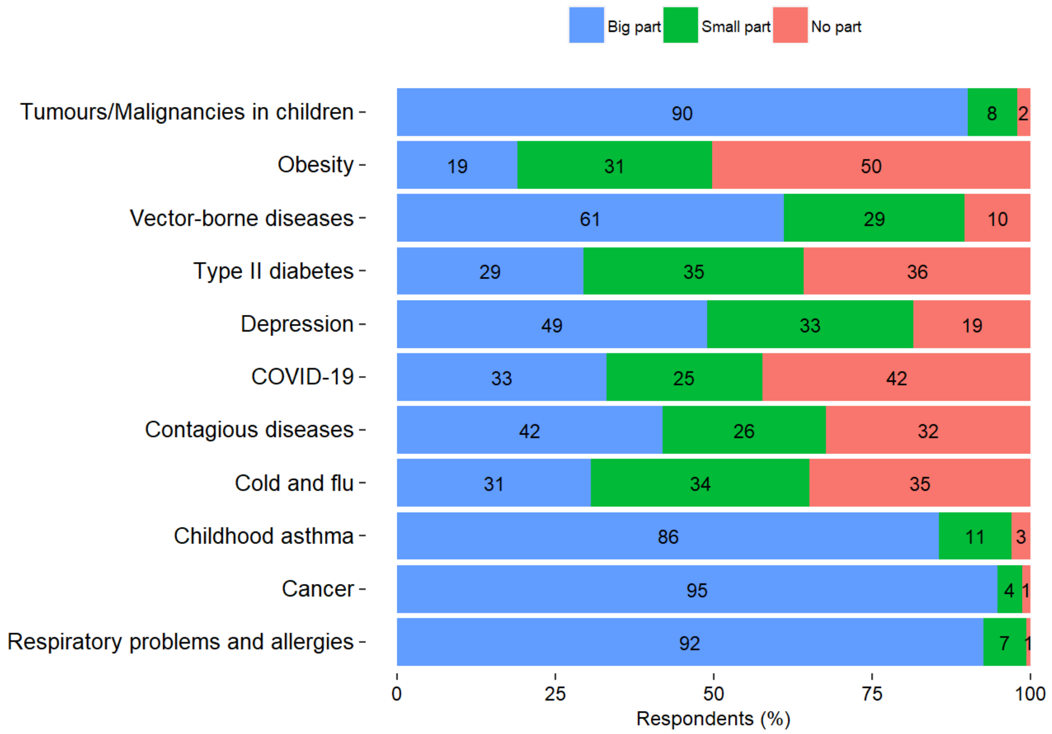


**Figure S17** Perceived part of environmental factors in diseases incidence (Question: Do you believe that environmental factors play a big part, a small part or no part in diseases incidence?)

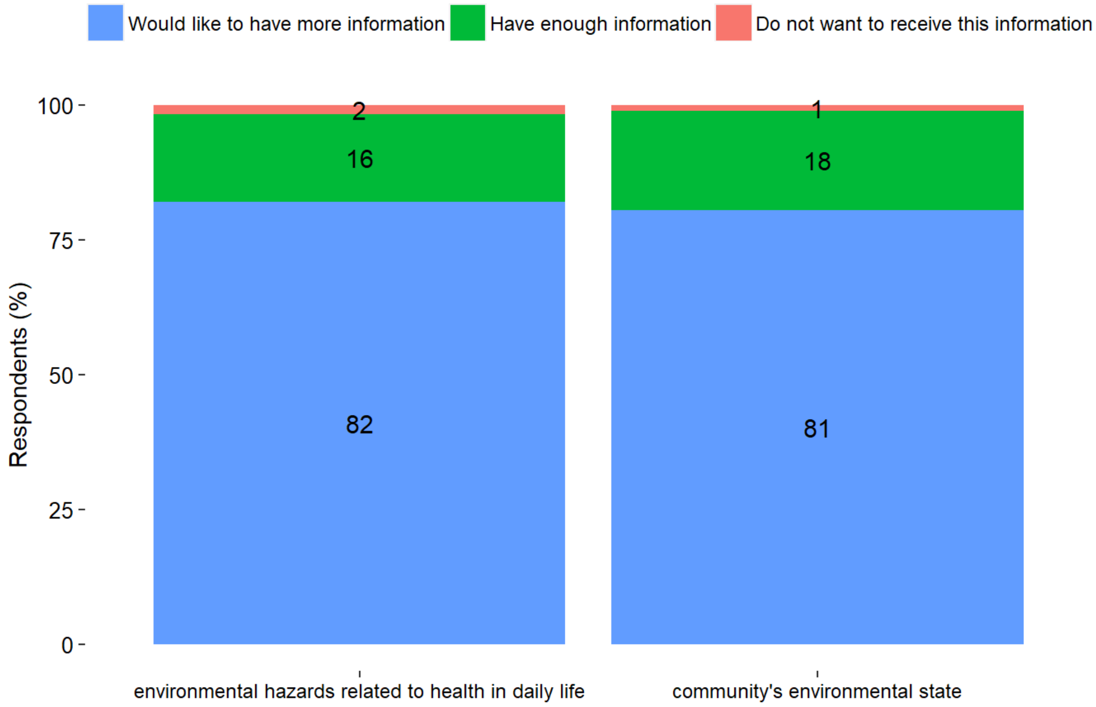


**Figure S18** Perceived information adequacy for environmental state of community and environmental hazards related to health (Question: Do you believe that you have enough information regarding the environmental state of your community and the environmental hazards that are related to your health in your everyday life?)

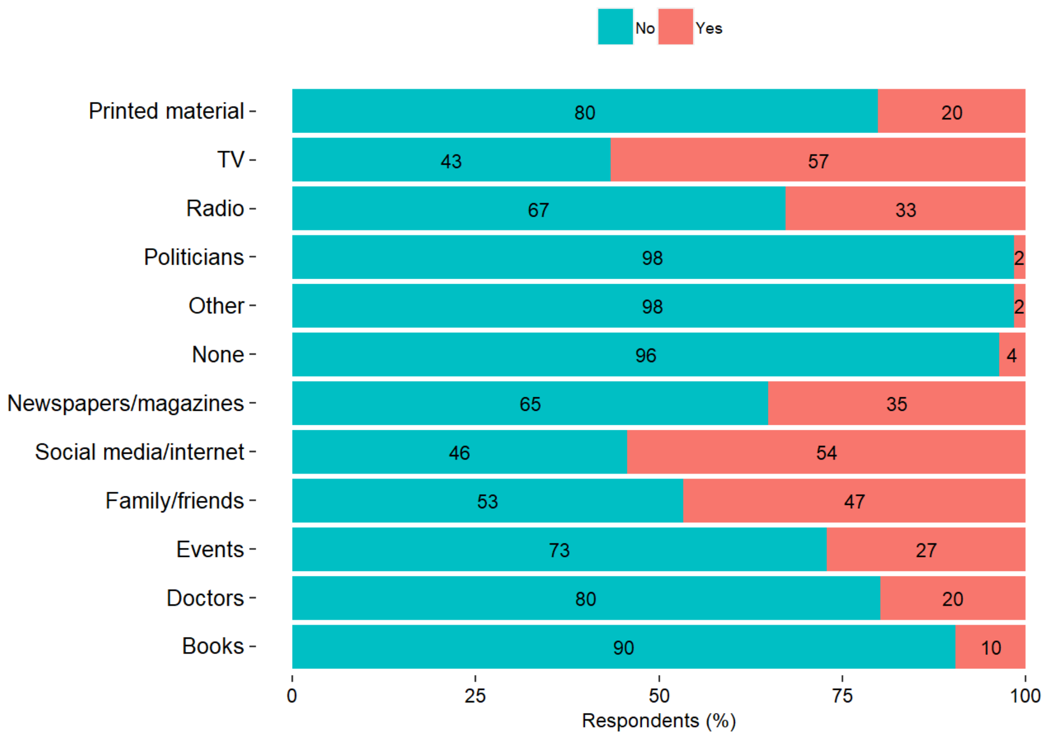


**Figure S19** Use of information sources for environmental hazards (Question: Which of the following ways / means do you use to receive information about environmental hazards?)

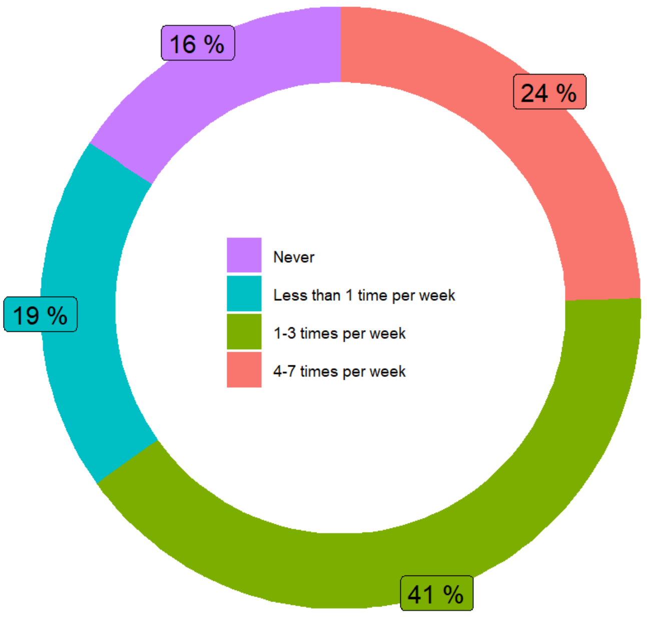


**Figure S20** Exercise frequency (Question: How often do you do physical exercise that includes movement for at least 30 minutes (e.g. brisk walking, running, aerobics, cycling) per day?

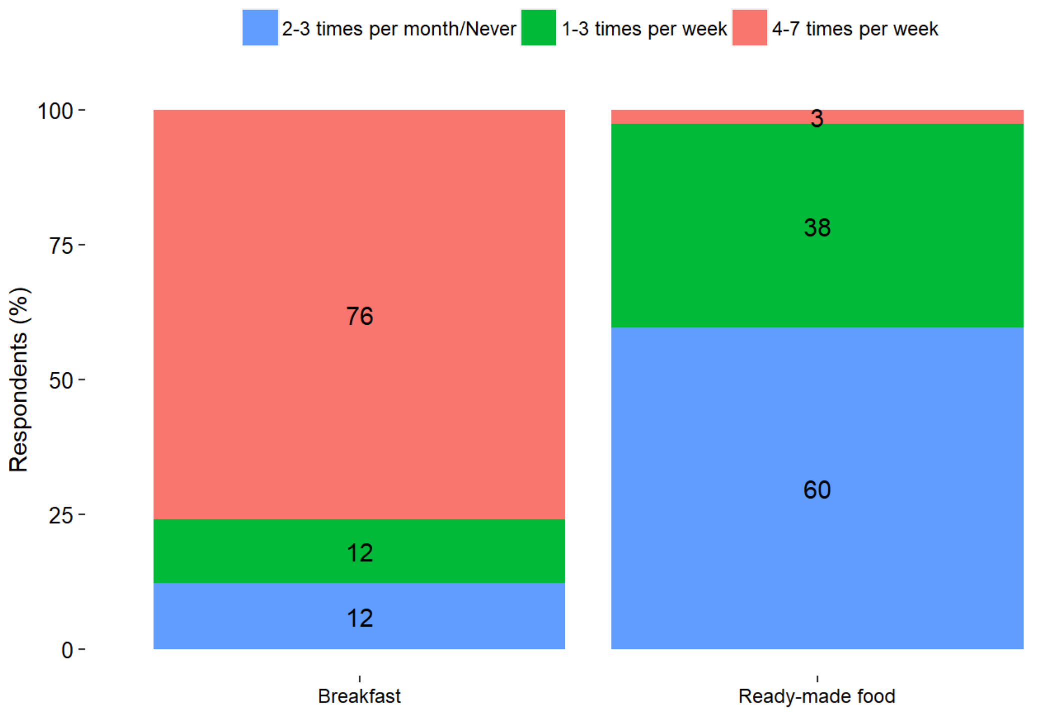


**Figure S21** Breakfast and ready-made food consumption frequency (Question: How often do you eat (i) breakfast (e.g. milk cereals, sandwiches, etc.)? (ii) ready-made food from outside (e.g. restaurant, barbecue, fast-food, etc.))

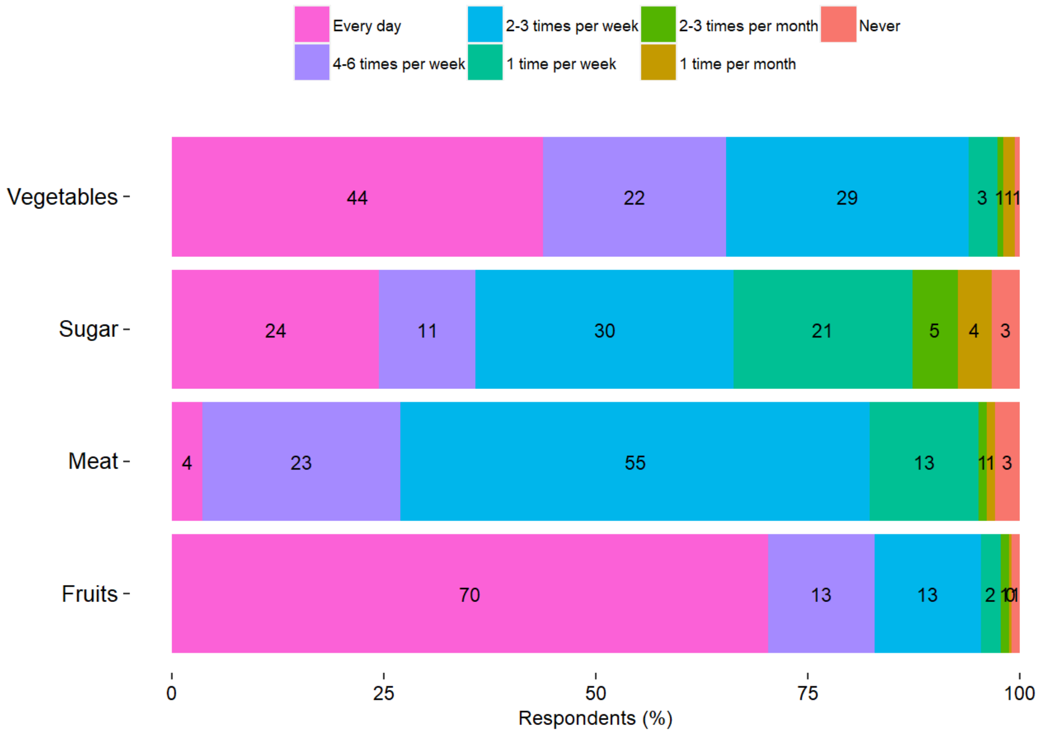


**Figure S22** Fruits, vegetables, meat and sugar consumption frequency (Question: How often do you eat (i) fruits? (ii) vegetables? (iii) meat? (iv) sugar (e.g. jam, honey, chocolate, biscuit, ice cream, cake)?)

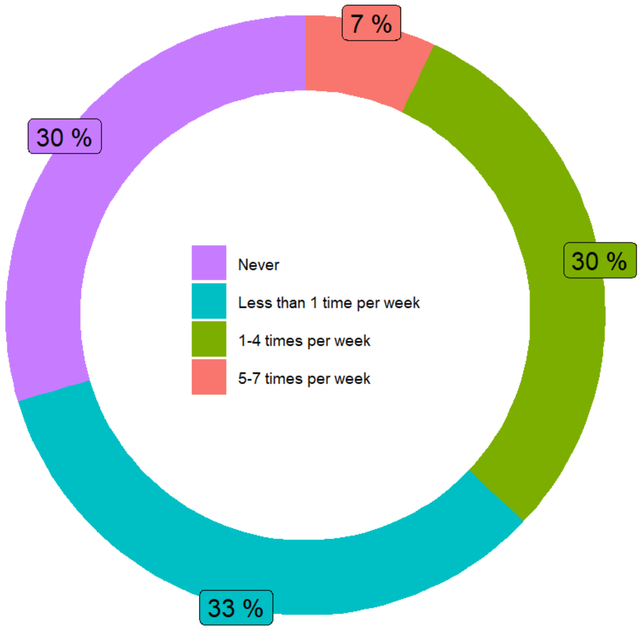


**Figure S23** Alcohol consumption frequency (Question: How often do you consume alcoholic beverages of any kind (portion: 1 glass of beer / wine, 1 unit of brandy / zivania / whiskey / ouzo / liqueur, cocktails with alcohol, breezers)?)

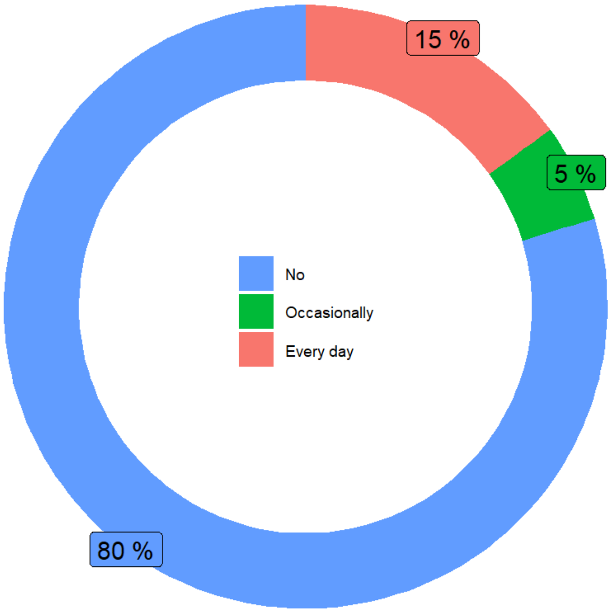


**Figure S24** Smoking frequency (Question: Are you smoking?)

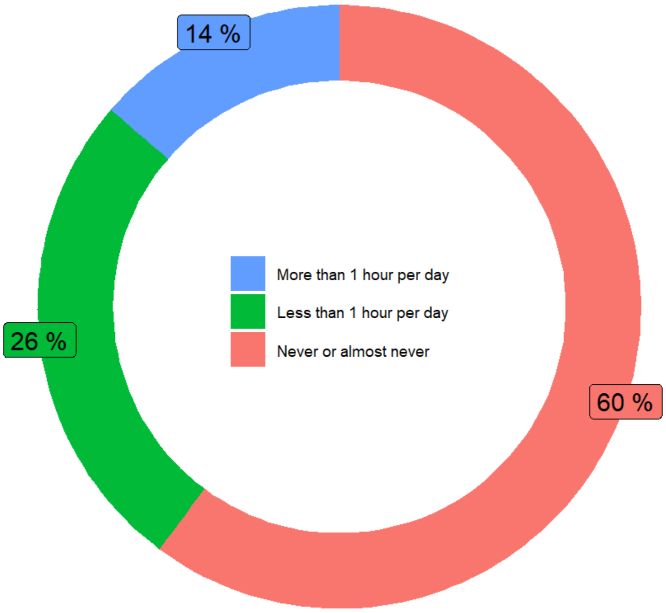


**Figure S25** Second-hand smoke exposure (Question: How often are you exposed to tobacco smoke from products that others smoke indoors e.g. at home, at work, in public places, in restaurants and on public transport?)

**Table S3** Environmental exposure and sensitivity

| | **Overall** | **Males** | **Females** |
|---|---|---|---|
| **n** | 304 | 144 | 153 |
| **Feel sick when exposed to smoke/fragrances/gasoline/pests etc. (%)** | 192 (63.2) | 77 (53.5) | 111 (72.5) |
| **Unable to tolerate or have adverse or allergic reactions to any drugs or medications, or medical/surgical/dental material or procedure (%)** | 67 (22.0) | 24 (16.7) | 40 (26.1) |
| **unable to tolerate or have adverse reactions to foods such as dairy products, wheat, corn, eggs, caffeine, alcoholic beverages, or food additives (%)** | 50 (16.4) | 22 (15.3) | 27 (17.6) |
| **No of "Yes" to above 3 questions (%)** | | | |
| 0 | 93 (30.6) | 58 (40.3) | 32 (20.9) |
| 1 | 141 (46.4) | 60 (41.7) | 80 (52.3) |
| 2 | 42 (13.8) | 15 (10.4) | 25 (16.3) |
| 3 | 28 ( 9.2) | 11 ( 7.6) | 16 (10.5) |
